# Molecule by Molecule Characterization of a Polymer Molecular Mass Distribution via Mass Photometry

**DOI:** 10.1002/anie.202518383

**Published:** 2026-03-19

**Authors:** Rachel Czerwinski, Anna L. Clayborn, Aisley Fleming, Andrew J. Boydston, Aaron H. Hoskins, Randall H. Goldsmith

**Affiliations:** ^1^ Department of Chemistry University of Wisconsin‐Madison Madison Wisconsin USA; ^2^ Department of Biochemistry University of Wisconsin‐Madison Madison Wisconsin USA

**Keywords:** Analytical Methods, Mass photometry, Polymers, Scattering, Single‐molecule

## Abstract

Polymer analysis tools form the backbone of modern polymer technology. However, traditional tools like size exclusion chromatography (SEC) fractionate based on hydrodynamic volume. This analysis assumes all molecules in a fraction are identical, an increasingly strained assumption for complex macromolecules. Importantly, characterization of the molecular mass distribution (MMD) is typically reduced to reporting several moments of the MMD, like number and weight‐averaged molecular masses, a significant loss of information. In contrast, a molecule‐by‐molecule elucidation of the full MMD would yield an extremely informative new perspective on macromolecule diversity, while eliminating limitations and time commitment associated with fractionation. Here, we show that mass photometry (MP), whereby the interferometric scattering signal of individual molecules is correlated with mass, of high‐molecular weight polyethylene oxide (PEO) allows elucidation of the full MMD. In some cases, multiple subpopulations are directly revealed. This effort provides the first single‐molecule measurement of the MMD of a synthetic polymer, bypassing artifacts introduced by bulk‐scale measurements. Further, we show that the dispersity (Đ) can be precisely determined in a manner that is largely calibration‐free, allowing determination of Đ in functional polymers where monodisperse standards are unavailable. Integration of MP with SEC conspicuously shows how multiple populations are present in collected fractions.

## Graphical abstract

Mass photometry (MP) was utilized to achieve label‐free, molecule‐by‐molecule measurement of the full polymer molecular mass distribution (MMD) of a synthetic polymer without the need for sample fractionation. The ability to elucidate the full MMD in minutes, as opposed to having only specific moments of the MMD acquired in hours via chromatography, has major potential for improving polymer characterization.

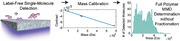

## 1 Introduction

A polymer's molecular mass distribution (MMD) is a fundamental characteristic of the inherently inhomogeneous polymer population and significantly influences the macroscopic physical properties [1, 2]. Consequently, a variety of methods have been developed to characterize polymer MMD, typically through determination of the number (*M*_n_) and weight (*M*_w_) averaged molecular weights,

(1) $$\;M_{n}=\frac{\sum M_{i}N_{i}}{\sum N_{i}}\;$$

(2) $$\;M_{w}=\frac{\sum M_{i}^{2}N_{i}}{\sum M_{i}N_{i}}\;$$

where *N*_i_ is the number of polymer chains with a particular mass, *M*_i_. Ideally, the summations in Equations (1) and (2) would count each individual polymer chain.

Size exclusion chromatography (SEC) and gel permeation chromatography (GPC) are powerful and widely used separation techniques that enable MMD characterization through calibration with polymer standards and a refractive index detector (RID), or in combination with static multi‐angle light scattering (MALS) [3, 4] and/or viscometry [5, 6] detectors. SEC separates molecules on the basis of their hydrodynamic volume: larger polymers with higher molecular weights generally elute first, while smaller species are retained by the column's pores and elute at longer times [7]. However, it is important to note that in both single and multi‐detector SEC, the summations in Equations (1) and (2) are not over individual polymer chains, but over collected fractions, where the many molecules accumulated together at each elution time are described by a single average value of molecular weight. Thus, SEC and its many variants are inherently ensemble‐averaged means of characterization. This limitation has major consequences as macromolecules with the same hydrodynamic volume but differing masses can coelute from an SEC column [8]. A true molecule‐by‐molecule means of characterization would eliminate issues arising from ensemble‐averaging over molecules with different properties. More importantly, a single‐molecule approach to determining polymer mass would provide a new characterization tool for polymer properties of unprecedented detail by making the complete MMD available rather than indirectly describing it though determination of the dispersity (Đ),

(3) $$ɡ\;=\frac{M_{w}}{M_{n}}\;$$

Single‐molecule microscopy and spectroscopy studies are known for their utility in revealing heterogeneity in a molecular population and chemical behavior that is hidden in bulk measurements. Uses of single‐molecule microscopy span studies of biological structure and dynamics [9, 10, 11, 12, 13], catalysis [14, 15, 16, 17, 18, 19, 20, 21], and include applications in the characterization of non‐biological macromolecules [19, 20, 21, 22, 23, 24, 25, 26, 27, 28, 29]. However, most single‐molecule studies rely on fluorescence, which generally requires the covalent bonding of a fluorescent label [30]. While labelling is at times valuable for polymer analysis [22, 23, 31, 32, 33, 34, 35], the need for fluorescent labels has limited the desirability and applicability of single‐molecule studies for routine polymer characterization.

**FIGURE 1:**
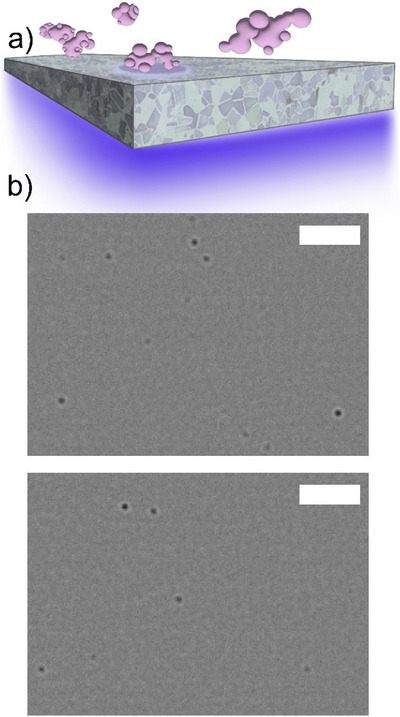
MP of PEO polymers. (a) Single polymer chains land on a glass surface. Light is incident from below, and weakly scattered light from the individual polymer molecules interferes with reflected light from the glass substrate, yielding a signal. (b) Two examples of MP images created by subtracting consecutively acquired frames. Dark spots are individually detected molecules. The range of contrast levels indicates different molecular weights. Acquired with S630, both scale bars 3 µm.

Interferometric scattering (iSCAT) microscopy, where a weakly scattered electric field is interfered with a much larger reflected electric field [36, 37, 38, 39, 40], offers a label‐free means of characterizing biomolecules and materials, including the widefield imaging of single molecules (Figure 1) [41, 42]. Mass photometry (MP) [37, 38] uses the detection principles of iSCAT for the label‐free determination of the masses of single biomolecules, including DNAs, RNAs, and proteins. In MP, signals originate from species binding to and unbinding from a partially reflective surface (Figure 1a) and are detected by comparing consecutively acquired images. These images are used to determine ratiometric contrast (the ratio of the molecular signal to the background, hereafter simply referred to as “contrast”), which scales linearly with molecular weight. Negative contrasts represent species landing on the partially reflective surface, while the rarer positive contrasts represent species departing. A more in‐depth discussion of MP's operating principles is offered in the Supporting Information. Due to its sensitivity and real‐time measurement capabilities, MP has been employed to examine a wide range of biomolecular assemblies [43, 44, 45, 46, 47]. Importantly, MP has recently been commercialized, widening access to the technique. However, MP has so far found only limited application in the characterization of non‐biological synthetic macromolecules [48].

The availability of label‐free methods [36, 38, 41, 42, 49, 50, 51, 52, 53, 54, 55, 56], including iSCAT microscopy and MP, opens new avenues for macromolecule characterization. Here, we explore the performance of MP for characterization of non‐biological macromolecules of non‐uniform molecular weight (i.e. synthetic polymers) and use high‐molecular weight standards of polyethylene oxide (PEO) as a model system. PEO was chosen because of the availability of high molecular weight standards and its water solubility. As shown below, we can elucidate the full MMD of a polymer sample via molecule‐by‐molecule analysis and calculate Đ values in qualitative agreement with other methods. Importantly, we also present the argument that determination of Đ can be performed in an essentially calibration‐free manner, a major attractive feature for polymer analysis where monodisperse calibration standards may be difficult to obtain. All measurements were acquired on the commercially available Refeyn Two^MP^.

There are however, readily apparent challenges to the application of MP to non‐biological macromolecules. PEO, which only contains oxygen as a heteroatom, is less polarizable (i.e. interacts less strongly with an electric field) than the biomolecules typically measured with MP. The contrast from a molecule is dependent on the molecule's polarizability, which can be thought of as a function of refractive index [57, 58]. Literature values for PEO's refractive index increment (dn/dc) measured under comparable conditions to those used in this work range from 0.134 to 0.139 mL/g [59], while the widely‐accepted value of 0.185 mL/g is used for proteins [60]. Importantly, the attribute of polymer distributions that makes them rich chemical systems, a near‐continuous distribution of molecular weights, is expected to add to the degree of difficulty for MP analysis. In contrast, most biomolecules have a well‐defined primary sequence of amino acid or nucleic acid monomers and are consequently highly monodisperse in their mass, even as more discrete heterogeneity exists in biomolecule assemblies. The significant increase in dispersity when moving from measurements of monodisperse proteins toward those of more broadly dispersed PEO will be a major factor in the analysis.

## 2 Results and Discussion

### 2.1 PEO Detection and Calibration of the Mass Photometer

Individual polymer chains of PEO are easily visible (Figure 1b), and their contrasts can be extracted. Calibration of the MP response against a series of PEO standards is a critical next step. The contrast value generated by a molecule landing or departing from a surface scales linearly with the mass of the scatterer [37, 38]. Importantly, contrast values are largely independent of macromolecular conformation and the orientation with which they land provided that the absolute macromolecular size remains well below the diffraction limit and circularly polarized excitation is used. This feature has been verified over a range of biomacromolecules of varying order and anisotropy [61]. For an MP calibration, samples of known mass are measured so the mass‐contrast linearity can be applied to determine mass from the contrasts of unknown species. Seven PEO standards were investigated for use in calibration, labelled by their listed *M*_w_ in kDa as determined by SEC: S44, S70, S100, S230, S250, S630, and S1200 (Table 1).

**TABLE 1:** Reported mass averages and dispersity values for the PEO standards used.

Sample	*M*_w_ (SEC)	*M*_n_ (SEC)	*M*_p_ (SEC)	*M*_w_ (Light scattering)	*M*_v_ (Viscometry)	Đ
S1200	1,200,000	1,110,000	1,180,000	1,200,000	Not reported	1.08
S630	630,000	594,300	610,000	580,000	672,500	1.06
S250	246,600	236,100	260,700	243,200	258,800	1.04
S230	231,600	223,200	206,300	231,400	241,300	1.04
S100	101,200	97,600	104,700	100,400	100,000	1.04
S70	70,000	66,100	75,100	70,000	70,100	1.06
S44	44,000	41,600	46,600	43,800	43,900	1.06

Calibrations were made with PEO at approximately 1 nM in water. Unless otherwise stated, all measurements on the Refeyn Two^MP^ were performed in “Droplet Dilution Find Focus” (DDFF) mode using the large, 202.4 µm^2^, field of view. An important practice was keeping the sample concentration low, as a lower concentration of molecules over a larger area can produce more reliable statistics while avoiding artifacts due to the spatial proximity of individual molecules (as is recommended for biomolecules) [62]. Minor issues encountered when using different focusing modes are summarized in the Supporting Information.

**FIGURE 2:**
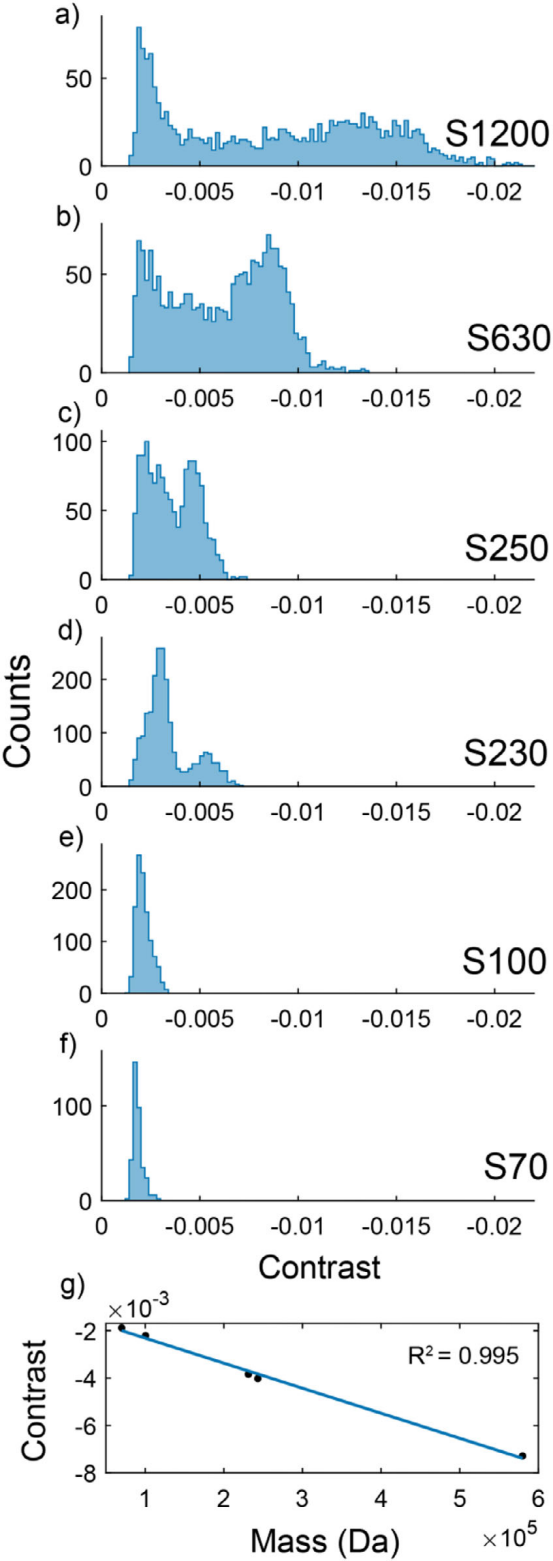
MP data of PEO standards (a–f), measured at 0.7 nM with DDFF and associated mass calibration comparing to *M*_w_ (LS) of PEO standards (g). S1200 is excluded from the calibration in (g).

The two smallest PEO samples (S44 and S70) were close to the instrumental mass detection limit of 30 kDa for proteins [62]. Lower limits of detection have been reported with use of machine learning in the data analysis [63], albeit for biomolecules with larger polarizability. Figure 2 does not include S44 as it was barely observable under these instrumental conditions. Despite consistent concentrations for all samples, the detectable counts from S70 in Figure 2 are notably lower than those of the larger molecular weight standards which is indicative of a decreased detection rate as the mass limit is approached. When carbonic anhydrase, a 30 kDa protein, was measured on MP, the resulting contrast values were comparable to the contrast values measured for S70, suggesting that, as these are theoretically the lowest resolvable contrasts, the mass detection threshold for PEO is indeed around 70 kDa. Further, measurement of contrasts of a similar magnitude for carbonic anhydrase as seen for S70 confirms that these small contrast values are reflective of polymer detection events rather than noise in the ratiometric background. Evidently, while MP can resolve biomolecules as small as 30 kDa, the less‐polarizable PEO has a higher mass threshold for detection.

Extending acquisition time past 120 s to collect more data and improve statistics for S70 and S44 was not helpful as the rate of observed events diminished over time, likely due to adsorption (Figure S6). Additionally, continuous illumination of the µL‐scale volume of solution has the potential to heat the sample, impacting the refractive index of the solution [64] and changing the observed scattering contrasts. PEO standards of 100 kDa were consistently detectable (see movie, Video S1) with total counts comparable to that of the higher mass samples. On the upper mass end, the S1200 polymer was also excluded from the calibration curve. Here, the point spread functions (PSFs) of each object began to appear distorted, as expected given that the object's physical dimensions are no longer negligible relative to the diffraction limit. Anisotropic fitting of the PSFs can potentially address this issue [61], but has not been attempted here.

The substantial inherent dispersity of even a PEO standard is evident in Figure 2. The broad distributions of contrasts for these samples were observed reproducibly (see Figure S13) and are discussed further below. The theoretical resolution limit for MP, defined as the full width at half maximum (FWHM) of the measured contrast, is fundamentally limited by Poisson (shot) noise from the high background [38, 65]. However, for measurements of PEO, background shot noise and polymer dispersity may both contribute to the broader FWHM of the observed peaks, with distributions sometimes exhibiting substantial deviations from the typical Gaussian shape. While the samples measured are low dispersity PEO standards, the histograms of contrast show some bimodal or multimodal character, especially for the highest molecular weights.

When performing an MP calibration for synthetic polymers, a major issue is what mass and what contrast should be used for comparison. In general, polymer standards are issued with statistically averaged masses and Đ values. For typical MP data on biomacromolecules with narrow dispersity, where the width of the largely Gaussian contrast distribution is determined by shot noise in the background, determining the mean value is a relatively straightforward procedure. But, even in a minimally disperse polymer standard, a diversity of molecular weights is present and not necessarily in a symmetric distribution. In Figure 2, only the two lowest mass samples display unimodal behavior with the higher mass samples exhibiting bimodal or even higher‐order distributions. One approach to calibration is to fit the contrast histograms with one or multiple Gaussians and choose the contrast value at the peak of the largest‐contrast Gaussian found (“peak picking”). However, since MP enables elucidation of the full MMD, one can calculate a weighted contrast average associated with *M*_n_, *M*_w_, or any other moment of the MMD (see Supporting Information for further discussion). We also explored using a protein ladder as a calibration standard, then converting to an effective PEO scale using the known ratio in refractive index increments (see Figure S2). Since the “peak picking” calibration yielded results that fluctuated from run to run, and use of a protein calibration resulted in calibrations that were reasonable at low masses but failed at higher masses, all subsequent discussion will entail determination of mass values using the full measured contrast distribution to calculate a weighted contrast average of the appropriate moment.

Figure 2g displays the calibration obtained when the contrast‐weighted contrast average value for each of the histograms shown in Figures 2a–f is correlated with its respective *M*_w_ measured via light scattering listed on the samples’ Certificates of Analysis (CoAs). Predictably, performing the calibration based on different moments of the MMD slightly altered the quality and parameters of the linear fit. Calibrations using the number average, viscosity average, and the weight average measured via SEC are shown in Figure S1. Using *M*_w_ measured via light scattering consistently maximized R^2^ and minimized the dispersity (discussed further below).

With *R* ^2^ values for the calibrations consistently above 0.98, calibrations appeared similar to those performed with monodisperse biomacromolecules. However, two differences are noticable. First, the slopes, (−1.1 x 10^−8^ in Figure 2g) were lower than with proteins, to a degree expected due to PEO's lower polarizability (see Supporting Information). Second, the y‐intercept values (−0.0013 in Figure 2g), were found to be elevated, roughly 10 times larger than those reported for monodisperse biomacromolecules [38, 66, 67]. This disparity may arise from a small systematic error in the reported masses of the polymer standards.

### MMD Distribution and Determination of Dispersity

**FIGURE 3:**
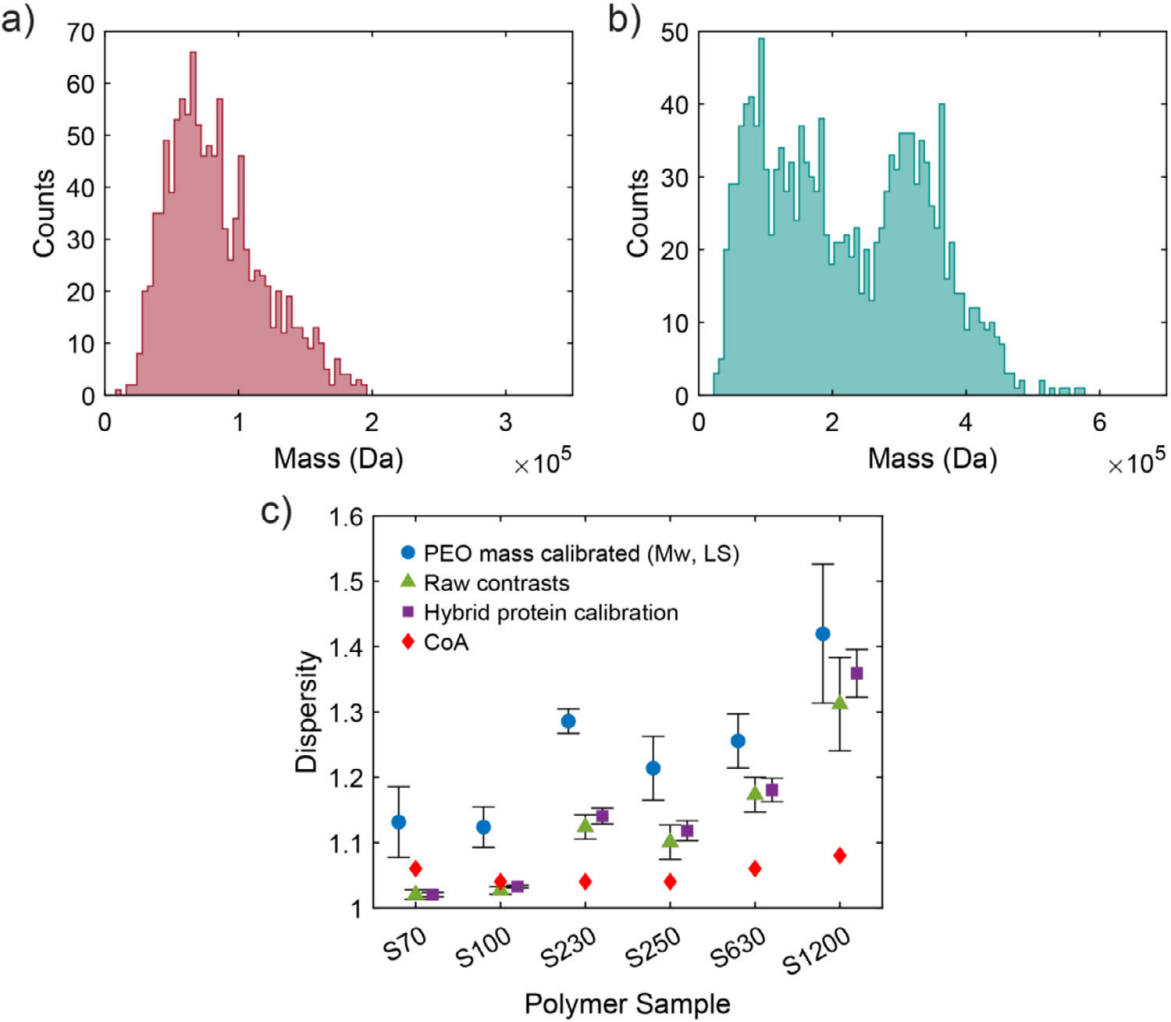
MMDs as determined by MP for S100 (a) and S250 (b) using *M*_w_ (LS) masses. (c) CoA (red diamonds) dispersity values are compared to those measured via MP using different calibration approaches: PEO mass calibration using *M*_w_ (LS) (blue circles), raw, uncalibrated contrasts (green triangles), and a hybrid protein calibration (purple squares). Points shown are an average of either 7 (PEO mass calibrated and raw contrasts) or 5 (hybrid protein calibration) dispersity values acquired from independent calibration runs. Error bars correspond to the standard deviation.

Armed with a calibrated MP signal, the complete MMD of the polymer samples can now be determined (Figure 3a, b). Importantly, this representation of the MMD was acquired without any type of fractionation and, because it was acquired molecule‐by‐molecule, has no binning artifacts resulting from polymers in the same elution volume being co‐analyzed to determine mass. The full MMD contains more information than traditional scalar representations of the MMD, such as the Đ value (Equation (3)) [2], since many uniquely shaped MMDs can be characterized by the same Đ. Building up the full MMD enables the visualization of sub‐populations, such as the low molecular weight sub‐population (centered at ∼1×10^5^ Da) in Figure 3b. While the populations of the lower mass chains are potentially elevated by PEO chain breakdown despite careful handling, the robust linearity of the calibrations involving the averaging of the entire contrast distribution suggests that these multimodal distributions are, to some extent, inherent to the polymer samples and not solely a result of chain scission.

Still, the comparison of Đ values is a straightforward way of juxtaposing the MP‐derived MMD with traditional measures of polymer dispersity. Both *M*_n_ and *M*_w_ can be directly computed from the MMD via the relations shown in Equations (1) and (2). We explored multiple ways of calculating Đ. One way directly uses the full PEO calibration shown in Figure 2 (Figure 3c, blue circles). However, we found that these determinations substantially overestimated Đ as compared to the manufacturer's CoA values (Figure 3c, red diamonds). Calibration with different empirically derived masses (Figure S1) or use of a peak picking calibration approach instead of weighted averages resulted in slightly different Đ values but generally displayed similar trends (Figure S3).

Importantly, inspection of Equations (1) and (2) offer another possible route, as Đ values are multiplicatively invariant to the underlying MMD (see Supporting Information), i.e. doubling every mass in the MMD results in an unaltered Đ. This property means that the value of Đ is invariant to the slope of the calibration. On the other hand, Đ is influenced by the y‐intercept, and increasing the magnitude of the typically observed negative intercept results in a monotonically increasing value of Đ. Thus, the calculation of Đ using uncalibrated contrasts sets a lower bound on Đ (Figure 3c, green triangles).

An intermediate option is accessible by performing a protein calibration just before the examination of the polymer. Use of a protein ladder yields a slope of −3.1×10^−8^ and y‐intercept of −0.00011, typical values for protein calibrations [38]. Importantly, the origin of the non‐zero y‐intercept is typically associated with minor instrumental artifacts such as incomplete background subtraction [63], suggesting proxy molecules (in this case the protein ladder, with known monodisperse masses) could be used to estimate the y‐intercept for the PEO sample, a practice consistent with other literature reports of transferring y‐intercepts between samples [67]. Uncalibrated contrast values can then be corrected using the y‐intercept from the protein calibration and subsequently used to calculate Đ. The results of this method are also shown in Figure 3c (purple squares).

Whereas the PEO‐only calibration is seen to vary widely between runs while overestimating Đ, likely because of deviations in the systematically large y‐intercept, use of the contrasts‐only determination of Đ (green triangles) or the hybrid approach (purple squares) with the protein‐derived y‐intercept result in repeatable values of Đ that are qualitatively similar to those reported in the CoA. The determination of Đ for S100 is particularly accurate, yielding Đ = 1.03, as compared to the CoA value of Đ = 1.04. Thus, MP can provide a widely reported metric of polymer properties without fractionation, with six polymer samples analyzed in only ∼20 min in total, as compared to the hours required for an SEC measurement (see Supporting Information). Importantly, determination of Đ does not require calibration with the polymer of interest, a feature which brings major advantages in practicality. Deviations in Đ values for S70 and S1200 likely originate in factors discussed above, with very small chains falling below the mass threshold for S70 resulting in a slightly underestimated Đ, and distortion of PSFs or chain fragmentation for S1200 resulting in an overestimated Đ.

### 2.2 Resolution of Peaks in Mixtures

**FIGURE 4:**
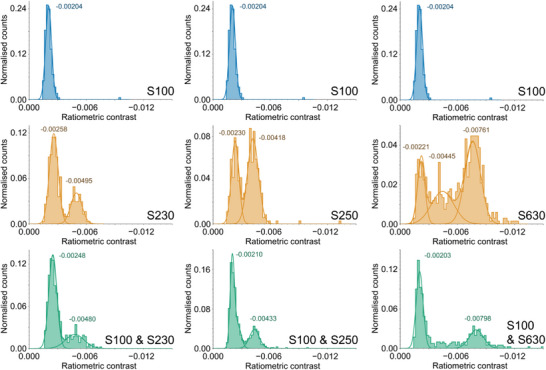
Resolution of multiple peaks from samples of mixed polymer standards via MP. For mixtures, each polymer was kept at 0.075 nM for a total polymer chain concentration of 0.15 nM. Collected using DDFF. Large contrast peak (−0.00761) in S630 histogram (middle right) has adjacent bins of equal height and is not cut off.

A viable polymer characterization method must resolve mixtures of polymers over a wide range of masses. For the largely homogenous, discrete biomolecules for which MP is typically employed, samples very close together in mass can be distinguished, with resolution up to 19 kDa for streptavidin (a 52 kDa tetrameric protein) and the achievable resolution decreasing at higher molecular weights [38]. As the dispersity of PEO increases peak width, samples need to be farther apart in mass to be distinguishable. To assess the ability of MP to resolve mixtures over a larger range of molecular weights, the standards used for the calibration in Figure 2 were used to form binary mixtures of consistent molarities (Figure 4). In Figure 4, contrast distributions are shown with Gaussian fits to aid in visualization of the populations present in each of the mixtures.

The lower bound of the Refeyn Two^MP^’s recommended working concentrations (0.1‐100 nM) [62] was most effective for the resolution of mixtures of PEO samples. Analysis at high dilution reduced the prevalence of outlier masses that contribute to overlap between peaks, though the lower count rate also reduced the mass precision to due to lower measurement statistics. Additional details on the effect of concentration on peak shape can be found in Figures S4 and S5 and is discussed in the Supporting Information.

Samples approximately 100 kDa apart were qualitatively resolvable (S230 and S100, Figure 4 left column). S100 (Figure 4, top left) appeared as a single distinct peak with contrast of −0.00204, while S230 (Figure 4, middle left) appeared as a bimodal distribution with peaks at contrasts of −0.00258 and −0.00495. The mixture of S100 and S230 (Figure 4, bottom left) exhibits contrast peaks at −0.00248 and −0.00480. The lower amplitude contrast peak of these corresponds to both S100 and the low mass population of S230, while the higher amplitude contrast of these is the smaller high mass S230 population. All subsequent discussion of contrasts will refer to the contrast amplitude.

In the combined sample, the lower contrast peak is substantially enhanced due to the combined effect of the S100 molecules and the lower molecular weight S230 molecules. Similar behavior is observed for S100 (reproduced for ease of viewing, top center, contrast −0.00204) with S250 (Figure 4, center, contrast −0.00418 and approximately half, −0.00230), which, when mixed (bottom center), shows contrast peaks of −0.00210 (S100 and lower mass S250 population) and −0.00433 (higher mass S250 population). S630 (right middle) is a more complex distribution of masses, with a high mass population centered at a contrast of ‐0.00761. An intermediate and low mass population are also identified with the Gaussian fits found at −0.00445 and −0.00221. When mixed (bottom right) with S100 (reproduced top right), a peak at −0.00798 corresponds to high mass population of S630 while the peak at −0.00203 corresponds both to the lower mass populations of S630 and the S100 component. Other intermediate mass S630 chains are responsible for the sparsely populated bins between the two peaks. In all three cases, the addition of the S100 polymer substantially intensifies the lower amplitude contrast peak. When the full MMD is made apparent, overlapping sub‐populations, even in reported low Đ value samples, are increasingly likely to be observed.

### 2.3 Integration of MP and SEC

**FIGURE 5:**
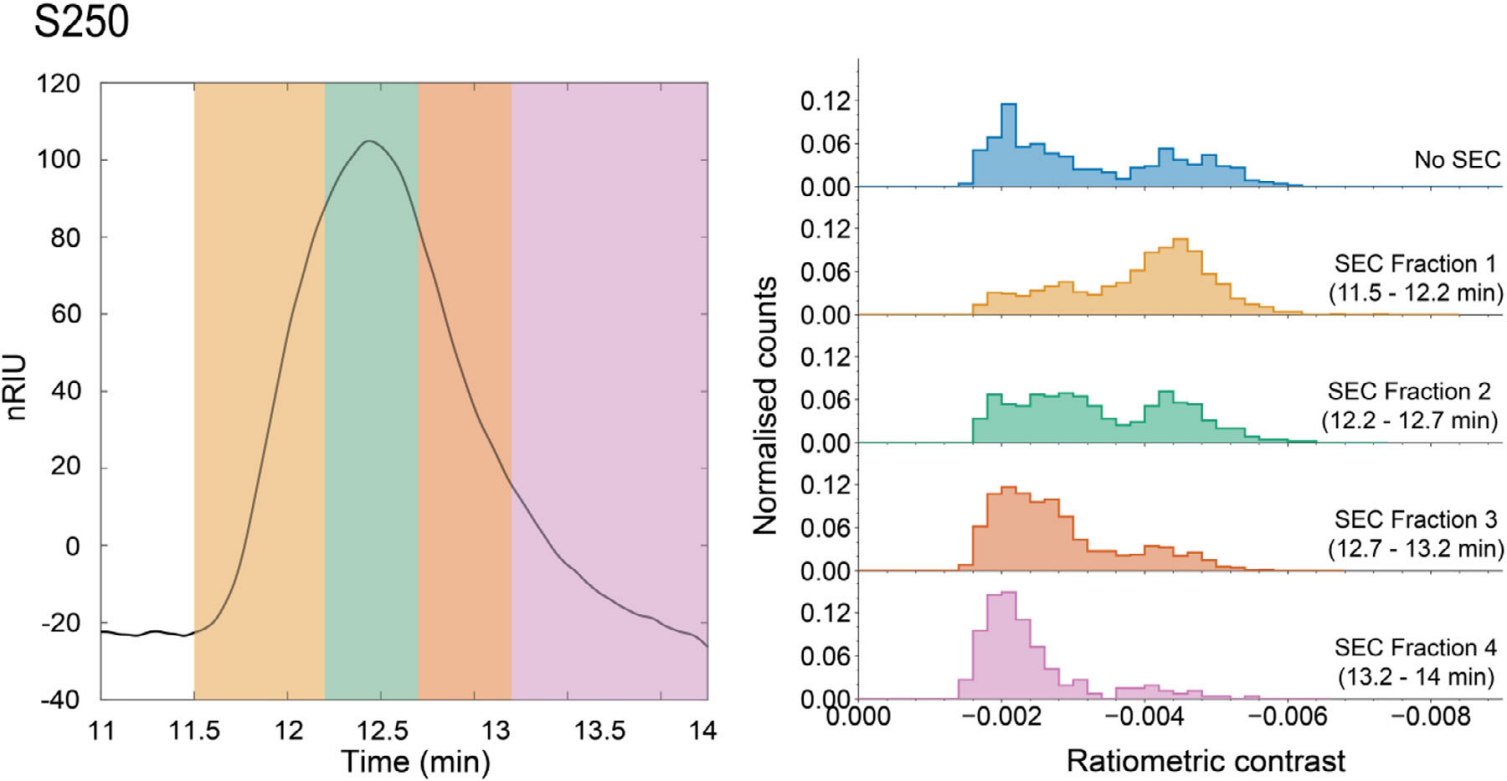
SEC of S250 with refractive index detection (left) with fractions of column output measured with MP (right). Large contrast population in unfractionated (blue) histogram is proportionally smaller than typical owing to minor precipitation of large chains during transport. MP histograms collected using DDFF.

Fractionation of a sample by SEC enables determination of mass and size parameters for each hydrodynamically separated fraction rather than batch averages, and is a staple of modern polymer analysis when combined with other bulk optical readouts [68]. Here, we explored the fractionation process by analyzing the output fractions via MP. A refractive index detector (RID) was used to monitor SEC fractionation of PEO standards in Table 1. SEC‐RID traces show that all the measured PEO samples exhibit a single, distinct peak (Figure 5 left, S8), despite the multipeaked‐MMDs visible via MP (Figures 2, 3, 4). Larger molecular weight standards elute faster, and the peaks notably broaden for more broadly dispersed larger molecular weight standards, as expected.

Given the polymer's MMDs, these SEC‐RID peaks do not represent homogeneous populations, and the character of the polymer eluting at the front of the peak is expected to be different from that eluting at the tail end, as verified and exploited by experiments where individual fractions are characterized by MALS, viscometry, or some other readout [68]. To see if MP can discern the differences in MMD for different parts of the peak, each peak was subdivided into multiple consecutive fractions which were collected in vials via the output of the column (Figure 5, left). We note that these fractions were collected by hand and consequently have larger volumes than in typical multiplexed SEC. The fractions were subsequently diluted and characterized via MP (Figure 5, right).

The MP data in Figure 5 shows the changing character of the PEO chains eluting off the SEC column over time. S250 is shown in Figure 5 and will be discussed as a representative example while SEC‐RID traces and MP of collected fractions for S100, S230, S630, and S1200 are shown in Figure S8. SEC‐RID for S250 showed a singular peak that eluted from approximately 11.5‐14.0 min. The top (blue) histogram shows unfractionated S250 as analyzed via MP. It is a largely bimodal histogram with a broad, lower contrast population centered around −0.0024 and a higher contrast population centered around −0.0046. The next (yellow) histogram shows MP characterization of the first fraction from the SEC column, taken from 11.5 to 12.2 min. MP analysis here again shows a generally bimodal distribution but with a significantly higher population in the high contrast peak and a correspondingly lower population in the low contrast peak. The next SEC fraction (12.2‐12.7 min, third MP histogram, green) shows the same bimodal distribution but with approximately equal peak normalized counts across the two populations. In the third SEC fraction taken from 12.7‐13.2 min (fourth MP histogram, orange), the low contrast features are significantly more populated than the high contrast peak. By the fourth SEC fraction (13.2‐14.0 min, fifth MP histogram, pink) the high contrast population has largely disappeared, leaving a dominant low contrast feature centered at −0.0021 and a few sparsely populated bins around the contrast value of −0.004. The clear shift in the dominant population from the larger contrast (higher mass) bins toward the smaller contrast (lower mass) bins over the course of the polymer elution is expected since larger molecular weight polymers typically have larger hydrodynamic radii and are anticipated to be more weakly retained by the stationary phase and elute earlier. A reconstructed contrast probability distribution can also be created by using the SEC trace (Figure 5, left) to yield a series of normalized weights, which are then applied to the MP traces from the individual fractions. This reconstructed distribution is seen to qualitatively replicate the multimode structure of the “No SEC” distribution, as expected (see Figure S9).

While SEC‐RID of S250 (Figure 5, left), S230, S630, and S1200 (Figure S8, left) show the MMD as a single peak, MP of the same polymer samples on the same day are clearly multimodal (Figures 5 and S8, right). Not only does MP reveal the underlying molecular subpopulations within a single SEC peak without fractionation but also reveals that the subpopulations persist despite fractionation. While these fractions have substantially larger volume than those taken in a typical multiplexed SEC experiment, the conspicuous presence of multimodal character across nearly all sections of the SEC peak underscores the level of macromolecular complexity that is potentially overlooked in a bulk experiment. Indeed, the observation of multimodal behavior by SEC‐RID often requires advanced separation techniques such as two‐dimensional chromatography [69, 70, 71].

### 2.4 Using MP to Monitor Chain Breakdown

**FIGURE 6:**
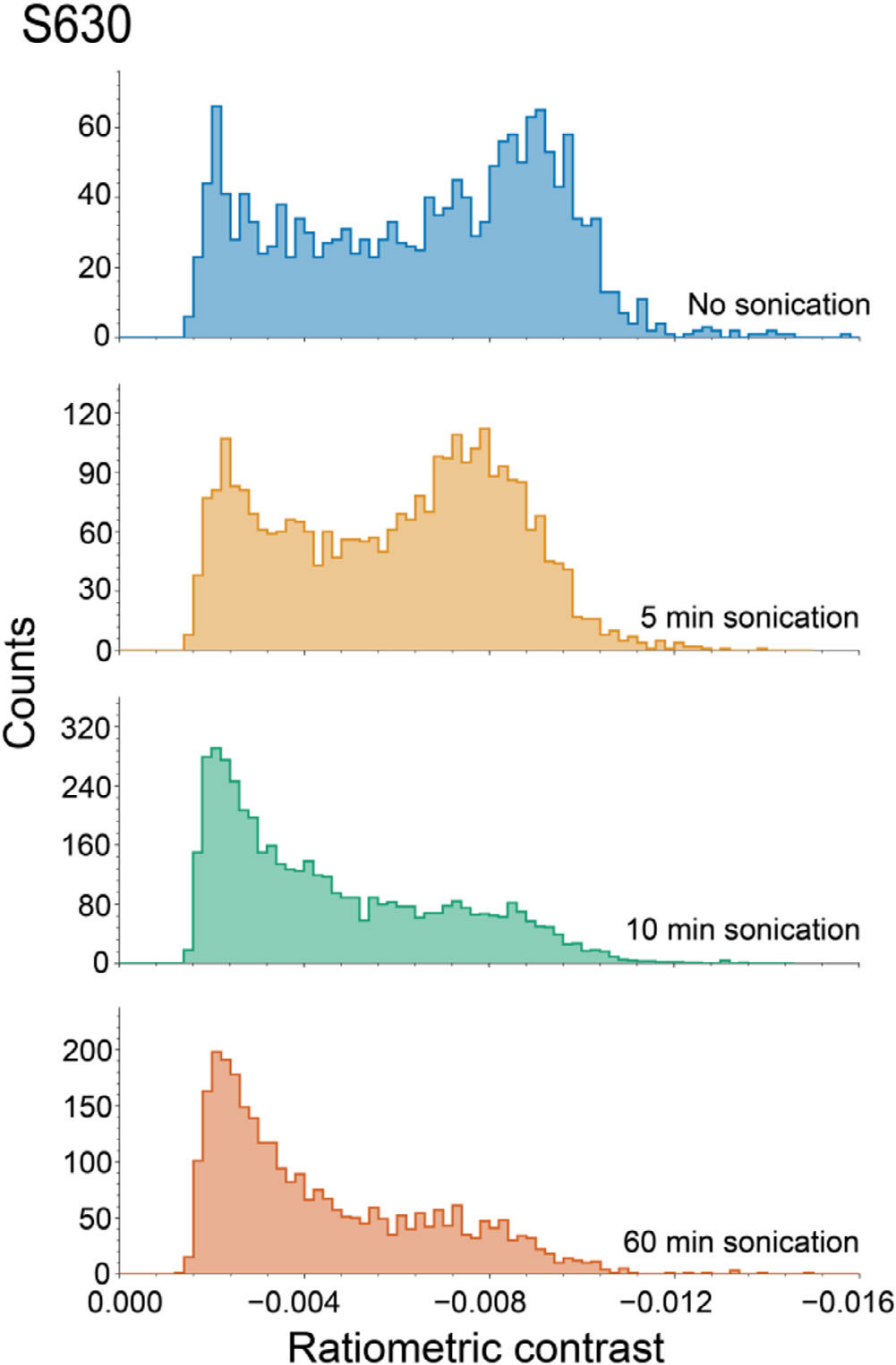
MP measurements of S630 sonicated for 0, 5, 10, and 60 min. Large FOV and DDFF were used.

It is well‐known that many polymer backbones, especially those with particularly high molecular weights, break down into smaller molecules when sonicated [72]. Sonication experiments were performed to assess MP's capability to quantify the change in the mass distributions of polymer standards due to chain scission. Samples each received 5, 10, and 60 min of sonication in a bath sonicator and their MP profiles at various sonication times were compared to MP data for the non‐sonicated control. The chain evolution for the S630 standard is displayed in Figure 6, and the other samples are shown in Figure S10. The most significant change in histogram shape occurs between 5 and 10 min of sonication. After 10 min of sonication, the population of the highest contrast events has noticeably decreased while the population of lower contrast events has increased, indicating the breakdown of higher mass chains. There is a subtle shift in toward lower contrasts that occurs during the first 5 min of sonication potentially due to the breakdown of the largest and, consequently, most fragile chains present.

With the exception of the sample sonicated for 60 min, the total counts observed for each sample increase as sonication time increases. A qualitative trend of this nature is expected as the samples are prepared in the same manner and thus the increase in molarity from the breakdown of larger chains into multiple smaller chains is not compensated for in sample preparation. While the shape of the histogram does not change significantly between 10 and 60 min of sonication, the decrease in counts suggests that breakdown still may be occurring but into pieces below the MP detection limit.

Across the entire 60 min sonication period, there is a clear decrease in population of the highest mass chains while the relative population of low mass chains centered approximately around −0.002 increases. This contrast corresponds to approximately one third of the contrast where the higher mass population is centered (−0.008). These fractional contrast values indicate that the large polymer backbones tended to break into several large pieces, behavior consistent with expectations [72, 73, 74, 75]. If instead there had been a tendency for chains of any given length to lose monomers from their ends, the distribution would have instead retained its general shape, and the peaks would simply be shifted to smaller contrast values.

Sonication‐induced chain scission of S230 was further investigated by SEC‐RID and subsequent MP of collected SEC fractions as shown in Figure S11. In addition to a conspicuous shift of the SEC peak toward longer retention times as sonication time increased, the observed population present in early timepoint fractions fell while the observed population in the later fractions rose. As in Figure 6, the polymer chains tended to break into several large pieces with contrasts equal to a whole number quotient of the full chain contrast.

## 3 Discussion

MP for MMD characterization is a powerful new tool for non‐biological polymer characterization. Molecular weight information from SEC, which intrinsically separates by the hydrodynamic volume can be confounded by surface interactions or branching [68]. The combination of SEC and MALS, a staple of modern polymer characterization, can only return accurate *M*_n_ values if the fractionation yields perfectly monodisperse aliquots, which is challenging for all but the simplest of polymers [68]. Conversely, MP is a fractionation‐free means of characterizing the full MMD and does not impose any requirements on the mapping between hydrodynamic properties and mass. Instead, the mass of each polymer molecule in the distribution is read out directly. The surface may alter the conformation of the macromolecule relative to its solution‐phase conformation, but the mass readout is unaffected [61]. In this manner, as shown in Figure 3a and b, key features of the MMD, such as shape or distinct subpopulations, are easily quantified. Knowledge of the full MMD is poised to have a major impact on how polymer scientists quantify their materials by allowing for potential correlation of macroscopic properties with subtle differences in MMD shape or minority polymer sub‐populations [2].

The lack of fractionation has substantial practical implications. Many of the most promising functional polymers are highly charged (polyelectrolytes), with applications from microelectronics to energy storage, water purification/separation, solar energy, surfaces, and drug delivery. However, charged polymers are extremely difficult to characterize via traditional tools [76], with the difficulty increasing with molecular weight and charge density. SEC fails because of strong interactions with the stationary phase and aggregation, which can at best skew the determined molecular weights and in many cases destroys the column. MP can potentially allow a fractionation‐free method of determining MMD in charged polymers, with substantial applications in polymer physics and materials. Importantly, difficult‐to‐produce narrow‐dispersity calibration targets of the same polymer composition may not be needed for determination of Đ since Đ can be determined without polymer calibration, removing a major impediment to adoption. This feature derives from the multiplicative invariance of Đ (see Supporting Information). Determining an absolute MMD distribution would still require calibration.

There are other practical advantages. The absence of the need for SEC fractionation makes MMD characterization substantially faster than SEC (discussed further in Supporting Information), potentially >10 times faster depending on the SEC retention time. MP is also easily accessible in a benchtop configuration. Low concentrations (<nM) and volumes of material (<10 µL) are required, enabling application to precious samples or low‐abundance populations. Application to branched polymers seems particularly appealing since such polymers frequently confound SEC analysis [68]. In applications where it is desirable to simultaneously measure multiple quantities, MP can be implemented after fractionation via SEC, allowing MP to fully integrate itself into the existing toolkit of polymer analysis, as shown in Figure 5. Additionally, light scattering [3] and intrinsic viscosity [5] detectors yield signals proportional to different moments of the MMD (the weight average and viscosity average, respectively), but these moments have increasingly disparate sensitivities to the low and high molar mass components of a MMD. Knowledge of the full MMD potentially reduces this bias toward specific molecular populations.

Going forward, a key activity will be more fully benchmarking MP against existing methods. As shown in Figure 3c, MP is capable of returning the same Đ as SEC analysis, as with the S100 sample, but in other cases provides a different Đ value. A logical question is then, what is the true Đ value? Any Đ value determined via SEC relies both on calibration via other SEC standards and the approximations at the foundation of modern SEC, which include the simple one‐to‐one mapping between hydrodynamic volume and mass, the inertness of the stationary phase [77], and depending on the standards used, the accuracy of universal calibration [78, 79, 80]. PEO, in particular, is known to be difficult to produce at low Đ values. Elucidation of the full MMD of the carefully handled PEO standards reveals significant populations of approximately half mass chains that are seemingly at odds with reported low Đ values. Further, examination of newly procured standards of the same lot number showed the same multimodal distribution of peaks (Figure S12). While it is clear when MP is grossly overestimating Đ, such as when the individual observed PSFs are anisotropic, when the returned Đ is somewhat elevated but qualitatively similar to other methods, it is unclear which value represents the true value.

The ideal way to benchmark MP would be to use extremely low Đ standards. Such standards exist for many polymer types [81], but few of these standards both possess high enough molecular weight for MP analysis and are soluble in water, the intended solvent for existing commercial MP systems. Due to the difficulty of maintaining living polymerizations in aqueous environments, water soluble synthetic polymers tend to have larger values of Đ. MP is easily calibrated against sequence‐specific biopolymers, but the mapping of contrast ratio between these biomolecules and synthetic polymers is not easily known, particularly when there is uncertainty in the value of the appropriate refractive index at the single‐molecule level [49]. Use of ratios of refractive index increments appears to allow coarse conversion that only works for low MWs (Figure S2). Appropriate calibration standards will need to be identified, particularly when absolute masses for the MMD distribution are desired.

Adaption of MP for non‐aqueous samples will also be necessary for many polymer systems. There is nothing in the physical mechanism of MP that precludes operation in non‐aqueous solvents, though refractive index contrast will be diminished (see Supporting Information), leading to higher minimum‐detectable molecular weights. Indeed, use of iSCAT in non‐aqueous solvents has already been demonstrated in home‐built systems [82, 83, 84]. In many cases, comparable sensitivity can be achieved by using solvents that maximize refractive index contrast. Pushing towards more widespread adoption of MP for synthetic polymer analysis necessitates further instrumentation and method development focused on increasing the sensitivity to the relatively small refractive index contrasts that may exist between non‐aqueous polymers and the nonpolar solvents they require and handling of those nonpolar solvents. Machine learning approaches have recently been employed to reduce MP's detection limit for biomolecules [63] and could assist in lowering the mass detection limit for aqueous polymers and increasing sensitivity.

Other technical challenges remain as well. Differences in counting efficiency as a function of molecular weight are conspicuous, with mass values close to the detection limit (which appears to be 70 kDa for PEO but will be even higher if organic solvents are used and will also be a function of monomer structure) being clearly undercounted. Dynamic surface effects resulting in a temporally variable background speckle can play a major role for objects near the detection limit [61]. These issues will be particularly important in cases where weighted average contrast values are used for calibration, as mentioned above. Once above the detection limit, MP has demonstrated an impressively consistent response at different molecular weights [38], and it may be that early applications of this technology will focus on higher molecular weight polymers. Another issue is binding efficiency. If different members of the chemical ensemble possess different binding rates and equilibrium constants, the resulting MMDs may be biased. This effect is not expected to be significant for a chemically homogeneous polymer over a reasonably narrow molecular weight range but may play a role for polymers composed of more chemically diverse monomers or blocks. Another potential issue derives from the mapping between mass and contrast. If an ensemble contains macromolecules with monomers with very different polarizabilities, it will be difficult to deconvolute chemical structure from mass. As above, this is not an issue for chemically homogenous polymers. Further, this is also not an issue with macromolecules where most molecules contain the same average percent composition of monomers, even if a variety of monomers with different polarizabilities are present. In proteins, with 20 different monomers over a range of polarizabilities, accurate masses are consistently determined by MP because the average amino acid composition is constant. A final issue is the connection between concentration and acquisition time. Use of higher concentrations conspicuously distorted the derived MMDs (Figure S4), but use of lower concentrations limited the size of the data set. When evaluating a complex MMD, more data will likely be required. Increasing integration time is thus desirable and will require further technical augmentation because of adsorption and local heating issues.

## 4 Conclusion

Mass photometry (MP) enables optical, molecule‐by‐molecule determination of the MMD of a synthetic polymer without fractionation in a manner that requires substantially less time per sample than SEC. In some cases, multiple distinct subpopulations were conspicuously observed via MP in a single polymer sample even as characterization of the same sample by SEC yielded a single peak. Use of the MP‐derived MMD allowed determination of Đ values that were consistent with SEC‐determined CoA values when the MMD was well‐within the MP dynamic ranges. Importantly, Đ can be determined without calibration standards due to the invariance of Đ to the MP slope. MP was integrated with SEC to demonstrate the broad arrays of polymer molecular weights contained in individual SEC fractions. MP allowed easy tracking of molecular weight changes upon exposure of the polymer standards to sonication. With these demonstrations, MP, which is already commercialized, has the potential to join the toolkit of techniques for characterization of polymers by providing a fast, benchtop method for elucidation of the full MMD without fractionation. However, additional benchmarking with more monodisperse standards will be necessary for widespread adoption.

## Conflicts of Interest

The authors declare no conflict of interest.

## Supporting information




**Supporting File 1**: Supporting Information document: Detailed experimental procedures, alternative calibrations, concentration effects, acquisition time dependence, additional SEC results, additional sonication results, examination of stability of standards. Supporting Information movie: a video showing MP data acquisition for the S100 standard.

**Supporting File 2**: anie71808‐sup‐0002‐VideoS1.mp4.

## Data Availability

The data that support the findings of this study are available from the corresponding author upon reasonable request.
