# Supplementary material for: Molecule by Molecule Characterization of a Polymer Molecular Mass Distribution via Mass Photometry
Source: Angew Chem Int Ed Engl. 2026 Mar 19;65(18):e18383. doi: 10.1002/anie.202518383 (PMC13110764; doi:10.1002/anie.202518383)
Supplement: Supplementary file 1 — Supporting File 1: Supporting Information document: Detailed experimental procedures, alternative calibrations, concentration effects, acquisition time dependence, additional SEC results, additional sonication results, examination of stability of standards. Supporting Information movie: a video showing MP data acquisition for the S100 standard. [file ANIE-65-e18383-s002.pdf]

Supporting Information for

Molecule by Molecule Characterization of a Polymer Molecular Mass Distribution via Mass Photometry

Rachel Czerwinski,[a]† Anna L. Clayborn,[a]† Aisley Fleming,[a] Andrew J. Boydston,[a] Aaron H. Hoskins, [a], [b] Randall H. Goldsmith [a]*

---

[a] Dr. R. Czerwinski, A. L. Clayborn, A. Fleming, Prof. A. J. Boydston, Prof. A. H. Hoskins, Prof. R. H. Goldsmith
Department of Chemistry
University of Wisconsin-Madison
1101 University Ave., Madison, Wisconsin 53706, United States
E-mail: rhg@chem.wisc.edu

[b] Prof. A. H. Hoskins
Department of Biochemistry
University of Wisconsin-Madison
433 Babcock Dr., Madison, Wisconsin 53706, United States

†Denotes first co-authorship

Table of Contents

Principles of MP, Connection to Other Quantities 4
Experimental 6
Comparison of Analysis Time for SEC and MP 8
MP Weighted Average Calibrations 9
Figure S1. 9
Protein Calibration 11
Figure S2 11
Extended Dispersity Figure 13
Figure S3. 13
Multiplicative Invariance of Dispersity Proof 14
Effects of Concentration on MP Peak Shape 15
Figure S4. 15
Figure S5. 17
Effects of Increasing Acquisition Time 18
Figure S6. 18
Figure S7. 18
Effects of FOV and Autofocus Settings 19
Discussion of polymer breakdown at the surface in MP 20
Size Exclusion Chromatography 21
Figure S8. 22
Figure S9. 23
Sonication 24
Figure S10. 24
Sonication and Size Exclusion Chromatography 26
Figure S11. 27
Stability of Standards 28
Figure S12. 28

Measurement Reproducibility 29
  Figure S13. 29
Sensitivity in Non-polar Solvents 31
Histogram Counts and Fitting Quality 33
  Table S2. 33
  Table S1. 33

Principles of MP, Connection to Other Quantities

This section covers a brief review of Mass Photometry, MP, and the reader is directed to several other primary sources and reviews for a more detailed description.[1-7] Light scatters from a single sub-wavelength-sized object, like a molecule, yielding a weak scattered electric field ($\vec{E}_{scat}$). At the same time, a much larger reflected electric field is produced from the glass-air or glass-water interface ($\vec{E}_{ref}$) due to the glass substrate employed in optical microscopy. At the squared law detector, the detected intensity is a result of interference between both electric fields.

$$I_{det} = \left|\vec{E}_{det}\right|^2 = \left|\vec{E}_{scat} + \vec{E}_{ref}\right|^2 = \left|\vec{E}_{scat}\right|^2 + 2\vec{E}_{scat}\vec{E}_{ref} + \left|\vec{E}_{ref}\right|^2$$

Here, a phase factor for the middle term is omitted for clarity. $\left|\vec{E}_{scat}\right|^2$, which is directly detected in dark-field scattering, is typically too small to measure for a single molecule and can be considered negligible here. $\left|\vec{E}_{ref}\right|^2$ is massive but contains no information from the molecule. On the other hand, the middle term sees the scattered electric field boosted by the reference electric field. Thus, molecules are visible via the second term, which appears as a small signal on top of a massive background from $\left|\vec{E}_{ref}\right|^2$. Further, $\vec{E}_{scat}$ is proportional to the excess polarizability, α, which is itself proportional to volume, *V*. For a molecular system of constant density, *V* can be well-approximated as being linear with mass.[6] This proportionality is the foundation of MP, whereby the scattered signal, as read out via the middle term in the expansion above, can be used to determine mass. Thus,

$$\vec{E}_{scat} \propto \alpha \propto V \propto mass$$

The polarizability, α, is also linearly proportional to the refractive index increment dn/dc.[8] In this context, multi-angle light (static) scattering, MALS, and MP originate from the same light-matter interaction.[6] However, whereas MALS is well-capable of determining the mass, even the absolute mass, of a uniform, homogeneous solution of polymers, it is incapable of resolving mixtures without fractionation. In contrast, MP can determine relative masses (and with calibration, absolute masses) in a molecule-by-molecule manner, eliminating the need for fractionation.

It is customary to express the signal in MP as a ratiometric contrast, *C*, which is in turn directly proportional to $\vec{E}_{scat}$, and thus the mass:

$$C = \frac{I_{det} - I_{bkg}}{I_{bkg}} \propto \vec{E}_{scat}$$

where I_{bkg} is the background intensity without the molecule present. Operationally, distinguishing between I_{bkg} and I_{det} is performed temporally, with consecutive images subtracted to form a difference image. In this manner, objects that land in a particular region yield a negative *C*, whereas objects that leave yield a positive *C*. These *C* values form the basis of all presented data and calculations. To convert *C* to an absolute mass value, a calibration is necessary, whereby molecules of known mass are studied via MP to extract a linear calibration relation.

Experimental

PEO standards were purchased from Scientific Polymer Products, Inc. and used as received. Polymer was dispensed into vials by mass and allowed to dissolve without agitation over several hours to avoid the breakdown of the fragile high-molecular weight chains. Unless otherwise noted, initial polymer solutions were made in 18.2 mΩ Millipore water to concentrations of approximately 2 mg/mL, then further diluted to single-molecule concentrations the day of measurement.

Protein calibrations were performed with bovine serum albumin (66.5 kDa monomer; Sigma Aldrich, A7638-5G) and bovine thyroglobulin (660 kDa dimer; Sigma Aldrich, 609310). Carbonic anhydrase (30 kDa; MP Biomedicals, 0215387910) was utilized for detection limit assessment. All protein samples were diluted in filtered phosphate buffered saline from frozen stocks at approximately 1 mg/mL to single-molecule concentrations on the day of measurement.

Working concentrations for MP of PEO were 0.25 – 2.5 nM. High precision cover slips (thickness 1.5H, 24x50 mm, CG15KH1, Thorlabs) were used for MP after being washed with HPLC grade methanol and Millipore water and dried with filtered house nitrogen. Grace Bio Labs Inc. Culture Well gaskets (3mm x 1 mm) were used to isolate up to six samples on each cover slip. Unless otherwise specified, all MP histograms shown represent 120 seconds of data collection.

All mass photometry data was taken on a Refeyn Two^{MP} running Refeyn Acquire^{MP} software, version 2.5.0. MP data was processed with Refeyn Discover^{MP} version 2.5.0. Data is reported in this document as only the negative “arrivals” for ease of viewing. PEO was observed to attach to the glass coverslips substantially more often detachment events were observed, especially for the higher molecular weight species. Although seen infrequently, when encountered, counts coming from large aggregates and time periods where significant background fluctuation occurred were removed from the MP movies in the Refeyn Discover^{MP} software.

Size exclusion chromatography was performed on an Agilent 1260 HPLC equipped with a refractive index detector (Agilent 1260 Infinity II - G7162A). The column used for separation was Agilent SUPREMA, 3x10^4Å, 8 x 300 mm, 10 µm, which uses polyacrylate particles as a stationary phase. Samples were prepared for SEC at

concentrations of 2 mg/mL. An injection volume of 75 μL and a flow rate of 0.75 mL/min were used for this analysis. The continuous phase on the column was 8 mM sodium azide to prevent microbial growth. The column temperature during separation was 70°C, and the RID temperature during separation was 40°C. Both the column and RID were allowed to equilibrate to temperature overnight with the HPLC in solvent recycling mode. One or more negative-going system peaks appeared in all sample and blank runs. System peaks in all sample runs were identical to the blank run from that day.

For MP experiments of SEC fractions, a SEC injection was run with RID equipped to record the peak shape and decide at what time points to set fractions. This initial run was immediately followed by the injection of another aliquot of the sample, where the output of the column was collected into a vial containing 1 mL Millipore water rather than being sent to the RID. SEC fractions were further diluted to MP concentrations with Millipore water and measured via MP the same day.

Samples exposed to sonication received this treatment at 2 mg/mL concentration. Sonication was carried out with a Branson 2800 Ultrasonic Cleaner at room temperature. Over the sonication period, the bath temperature was allowed to rise. For all sonication experiments, an aliquot of solution was set aside before any sonication exposure and further aliquots were drawn after 5, 10, and 60 minutes of exposure.

Comparison of Analysis Time for SEC and MP

As mentioned in the main text, MP requires substantially less time per sample than SEC. Acquisition of data on a single polymer takes <2 minutes. When including calibration and imaging processing, measurement and analysis of a set of six polymer samples on MP can be completed in approximately 30 min. In contrast, running a single sample on SEC without calibration takes at least 20 minutes. When running six samples (without additional calibration of the column), including the needed flushing of columns between samples, at least 2.5 hours is required. Therefore, analysis via MP constitutes a significant decrease in measurement and analysis time from SEC, particularly for polymers requiring lengthy separation times on the column.

MP Weighted Average Calibrations

MP data for the PEO samples measured with large FOV and DDFF is shown in main text Figure 2. The calibration shown in Figure 2g (reproduced here as Figure S1b) correlates the contrast-weighted contrast average (C_w) for each sample with the M_w measured via light scattering reported on the Certificates of Analysis (CoA). The contrast-weighted contrast average is a direct analogy of M_w and is calculated for each PEO sample according to the equation shown below where C_i is each contrast value measured and N_i is the number of counts observed for that contrast value.

$$C_w = \frac{\sum C_i^2 N_i}{\sum C_i N_i}$$

Figure S1 also includes the calibrations relating the number and viscometric contrast weighted averages to the corresponding mass averages from the CoA. As mentioned in the main text S1200 was excluded from the calibrations as the PSFs appeared distorted, which can lead to lower than expected contrast values. Equations for calculating the viscometric (C_v) and number (C_n) contrasts averages are shown below. The variable ‘a’ is the Mark Houwink parameter which is related to a polymer’s shape in a specific solvent at a specific temperature. A literature value of 0.82

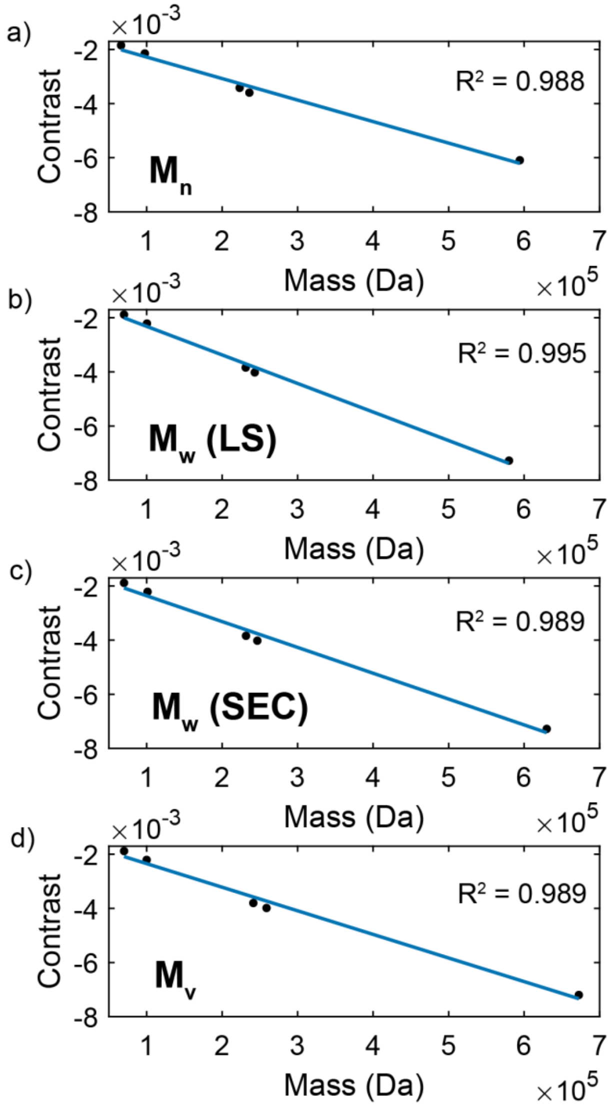

Figure S1. Mass calibrations relating weighted averages of measured contrasts of different moments to the corresponding mass moments from CoAs. Corresponding histograms and M_w (LS) calibration (reproduced here) shown in Figure 2.

for PEO in water at 308 K was used.[9] These equations are, as above, in direct analogy to M_n and M_v.

$$C_v = -\frac{\sum(-C_i)^{a+1}N_i}{(\sum(-C_i)N_i)^{\frac{1}{a}}}$$

$$C_n = \frac{\sum C_i N_i}{\sum N_i}$$

As discussed in the main text, use of the contrast-weighted average correlated with the M_w measured via light scattering consistently maximized the R^2 and minimized the calculated dispersity when the fit was applied to the contrast distributions. This is likely in part because the weighted average M_w (LS) calibration reproducibly had the lowest y-intercept values.

For all calibration and dispersity calculations, contrast values with a magnitude greater than the mean of the distribution plus 3 times the standard deviation were removed. On average, this filtering resulted in the removal of around 1% of contrasts with the max percentage of contrasts removed for any one sample being around 2% of the total contrasts. Eliminating these outliers avoids issues where a single extremely large scatterer (i.e. a piece of dust) skews the weighted contrast average values of which C_w is especially sensitive to the highest magnitude contrasts.

Protein Calibration

Figure S2 shows calibrations for a protein ladder (red) as well as a typical weighted average PEO calibration (blue) for the MP data shown in Figure 2 and protein data taken on the same day. The protein calibration was constructed from 60s MP movies taken of samples of bovine serum albumin (BSA) and bovine thyroglobulin in phosphate buffered saline. Peak contrasts from Gaussian fits of the resulting histograms were assigned to the BSA monomer (66.5 kDa), dimer (133 kDa), and trimer (199.5 kDa), and to the thyroglobulin monomer (330 kDa), dimer (660 kDa), and tetramer (1320 kDa). The protein calibration shown has a slope of -3.1 x 10^{-8}, a y-intercept of -0.00011, and an R^2 of 1.000.

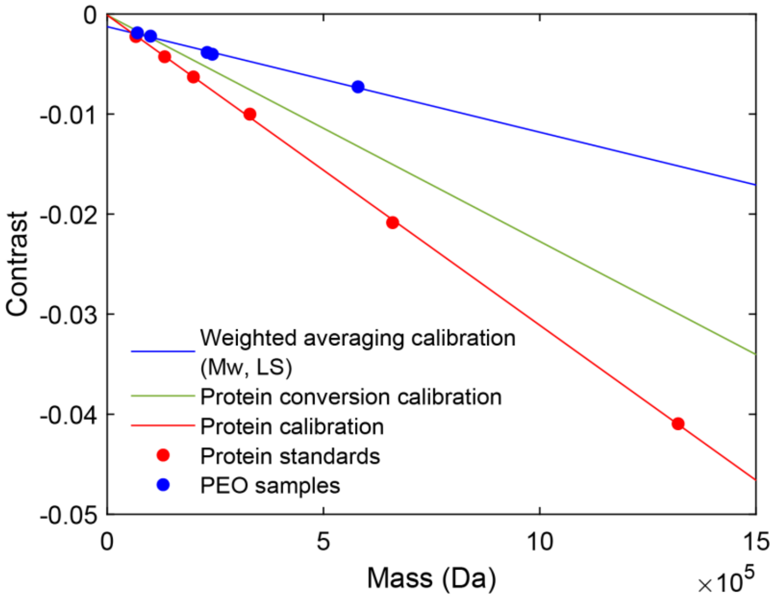

Figure S2. Protein (red), protein conversion (green), and weighted averaging PEO (blue) calibrations. Contrast weight averages shown with corresponding M_w (LS) values (blue circles) and peak contrasts for protein samples with corresponding masses (red circles).

As discussed in the "Principles of MP, connection the other quantities" section above, the contrast is linearly proportional to the excess polarizability, which in turn is linearly proportional to the dn/dc. Therefore, as the calibration slope is a function of the excess polarizability of the scatterer, the ratio of PEO dn/dc to that of protein can be employed to estimate the slope of a PEO calibration. Here we utilized the widely reported dn/dc value of 0.185 mL/g for proteins[10] and a value of 0.135 mL/g for PEO.[11] The green line in Figure S2 shows the result of multiplying the ratio of the PEO and protein dn/dc values by the slope obtained for the protein calibration. Here the y-intercept from the protein calibration is used unchanged. This 'protein conversion calibration' works reasonably well at low masses (weight contrast average values for S70 and S100 shown as blue dots). However, due to the significant differences between the weighted

averaging PEO slope (-1.1×10^{-8}, blue) and protein conversion slope (-2.4×10^{-8}, green) the calibration deviates dramatically from the PEO data at higher masses. This is potentially due to the inability of the bulk dn/dc values to capture subtleties in the polarizability of PEO and proteins across an extended mass range.

Extended Dispersity Figure

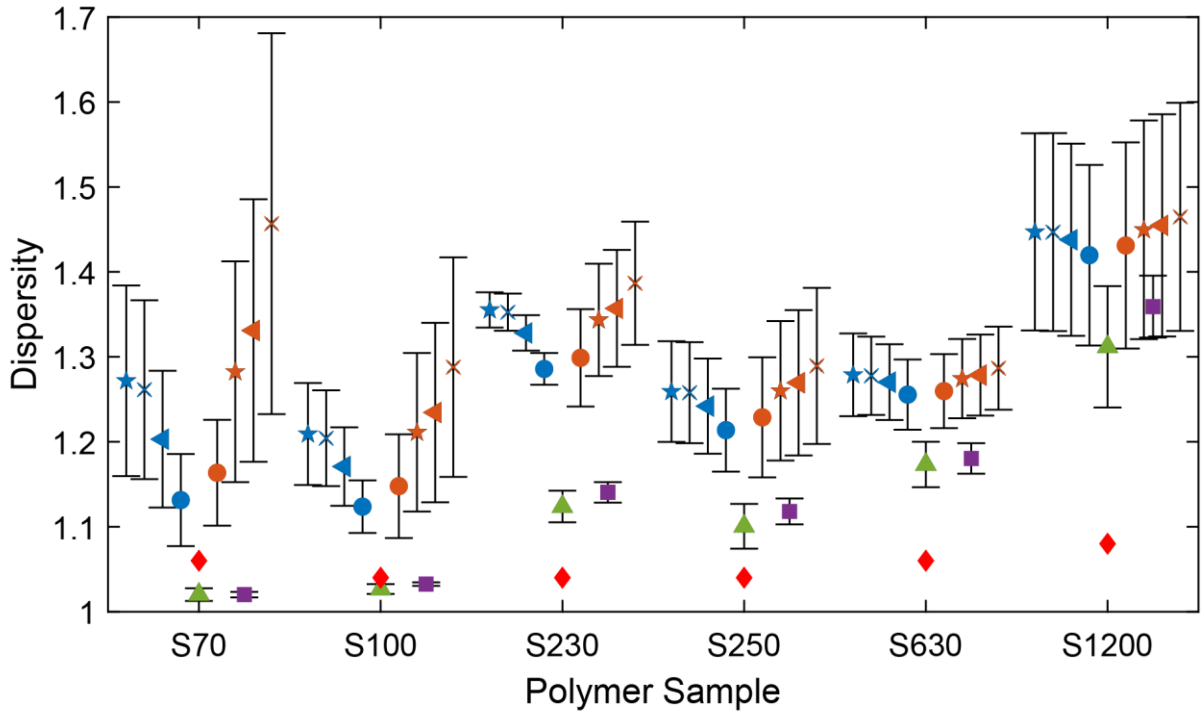

Figure S3. Certificates of Analysis (CoA) (red diamonds) dispersity values are compared to those measured via MP using multiple different calibration approaches. Blue circles, green triangles, and purple squares are the same as in Figure 3c. Other blue symbols correspond to different weighted averages corresponding to different types of masses. Orange symbols correspond to peak-picked calibrations.

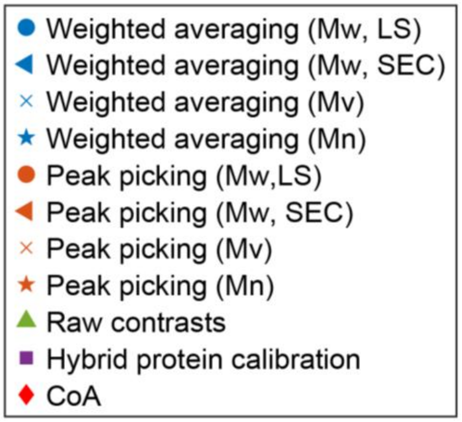

As described in the previous SI section, “MP Weighted Average Calibrations”, a variety of different mass definitions can be used for calibrations, and thus to calculate dispersity values. In addition, instead of performing weighted averages, one could also correlate the peak of the largest Gaussian component of the MMD with the various listed masses (a “peak-picked” calibration). Dispersity values calculated with these different definitions are shown in Figure S3.

Multiplicative Invariance of Dispersity Proof

Starting from equations (1,2),

$$M_n = \frac{\sum M_i N_i}{\sum N_i} \quad (1)$$

$$M_w = \frac{\sum M_i^2 N_i}{\sum M_i N_i} \quad (2)$$

The Dispersity, Đ, can be written as,

$$Đ = \frac{M_w}{M_n} = \frac{(\sum M_i^2 N_i)(\sum N_i)}{(\sum M_i N_i)^2}$$

Now, let's replace each Molar mass value, M_i, with that same Molar mass multiplied by a factor c, such that $M_i \rightarrow cM_i$. Then,

$$Đ = \frac{(\sum (cM_i)^2 N_i)(\sum N_i)}{(\sum cM_i N_i)^2} = \frac{\cancel{c^2}(\sum M_i^2 N_i)(\sum N_i)}{\cancel{c^2}(\sum M_i N_i)^2} = \frac{(\sum M_i^2 N_i)(\sum N_i)}{(\sum M_i N_i)^2}$$

Because the multiplicative factor, c, can be taken out of the summation, multiplication by a constant factor results in an unchanged value of Đ. Đ is multiplicatively invariant.

On the other hand, if a factor, h, is added to each M_I, such that $M_i \rightarrow M_i + h$, the value of Đ will change. Thus, Đ is not additively invariant.

Graphically, doubling the mass of the MMD center while also doubling its width does not change the value of Đ. But, doubling the mass of the MMD center while keeping the same width results in a different value of Đ.

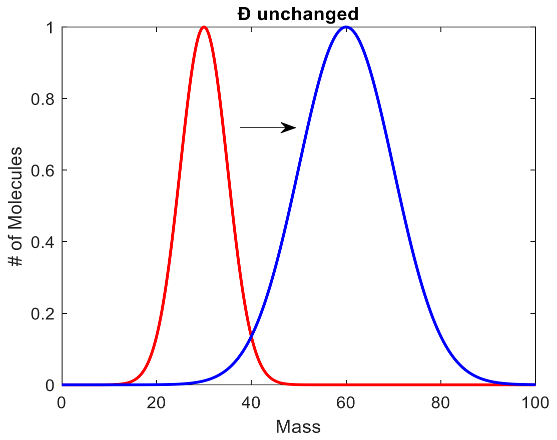

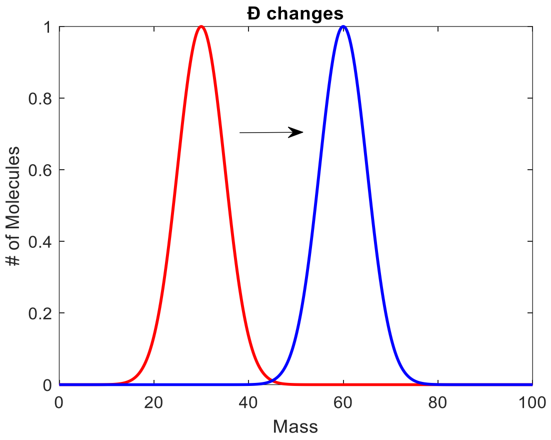

As a consequence, the Đ value is not a function of the mass photometry calibration slope (the *m* in *y=mx+b*), but is a function of the mass photometry calibration y-intercept (the *b* in *y=mx+b*).

Effects of Concentration on MP Peak Shape

As discussed in the main text, even the most narrowly distributed polymer standard will be more polydisperse than a protein with set primary sequence and very little variation due to mutation. The peaks measured on MP are therefore not only broadened by the resolution of the instrument, but also by their dispersity. Lower concentrations are used in MP to resolve mixtures of populations as decreased concentration limits the overlap of the shoulders of adjacent peaks which obscures resolution of distinct subpopulations. . To resolve the shape of the PEO peaks, the lower end of the working concentration range for MP was used for all experiments (0.1 nM – 2.5 nM).

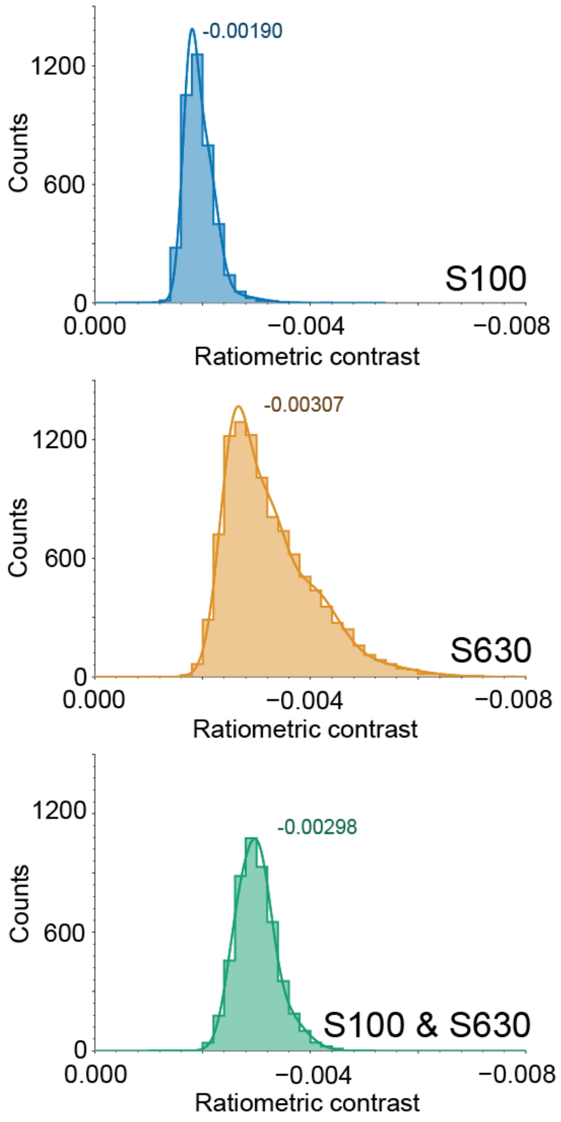

Figure S4. S100 (top), S630 (middle) and a mixture of both samples measured on MP at 15 nM overall concentration, with Large FOV and Buffer Free autofocus. 60 seconds data collected.

When polymer concentration was too high, a mixture of two standards would show only one feature on MP, with a peak contrast somewhere between the two values expected. Figure S4 exhibits this phenomenon, with MP data for 15 nM PEO samples in water. The top histogram, S100, shows a single peak with contrast of -0.00190, not dissimilar to the single Gausian behavior S100 exhibits at lower

concentrations. The middle histogram of Figure S4, however, shows a singular broad peak for S630, as opposed to the multimodal histograms this sample shows at lower concentrations. Additionally, its peak contrast is -0.00307, whereas in Figure 2b a contrast of -0.003 corresponds not to the center of a major population but rather the smaller intermediate population located in the valley of the histogram. . Combining the two samples at these high concentrations (Figure S4, bottom), resulted in not two separate peaks, but rather a singular peak set between the two samples, with a contrast of -0.00298. This perfectly illustrates the merging behavior of mixed samples at high concentrations, and explains the loss of S630's multimodal behavior in Figure S4. As the concentration of PEO is lowered to the working values of 0.25-2.5 nM used in this study, artifacts are decreased enough that an undistorted distribution of the sample can be visualized.

Other artifacts appear when examining mixtures under conditions of constant total mass (unlike Figures 4 and S4, which are at equal molarity of polymer chains). All single-component and multi-component mixtures shown in Figure S5 had an overall concentration of 7.5E-4 mg/mL (6.8 nM S100, 3.6 nM S230, 2.9 nM S250, and 1.2 nM S630) . Each column represents a different combination of samples, where the top (blue) sample and the middle (yellow) sample are combined in the bottom histogram (green). S100 presented as a singular peak with a contrast of -0.00185. When it was combined with other samples, their contrast values began to pull in closer to the center. In the leftmost column, for example, S230 when measured alone shows a small peak with ratiometric contrast of -0.00409 and a larger peak with contrast -0.00219. When combined with S100, MP shows peaks at -0.00170 and -0.00267, the peak representing the biggest chains receeding inward to a lower contrast value. Similarly in the middle column, S250's peak at -0.00338 shifts to -0.00243. In the rightmost column, S630's peaks of -0.00471 and -0.00643 are shifted to -0.00324 and -0.00586, respectively.

The dramatic inward shift in the ratiometric contrast from the individual component samples to the two-component mixtures can be partially attributed the significant diffrerence in molarity of the two components. In equimass mixtures with S230, S250, and S630, the S100 component is approximately 2 to 5.5 times more concentrated than the larger mass component. Therefore, events caused by the S100 chains dominate the

histogram with the larger contrast shoulder of the S100 peak adding with the meager populations of the large mass component to generate a peak at a much lower contrast value than the events from the large mass component alone generated. The S100 sample is simply too concentrated and obscures the signal from the higher mass components.

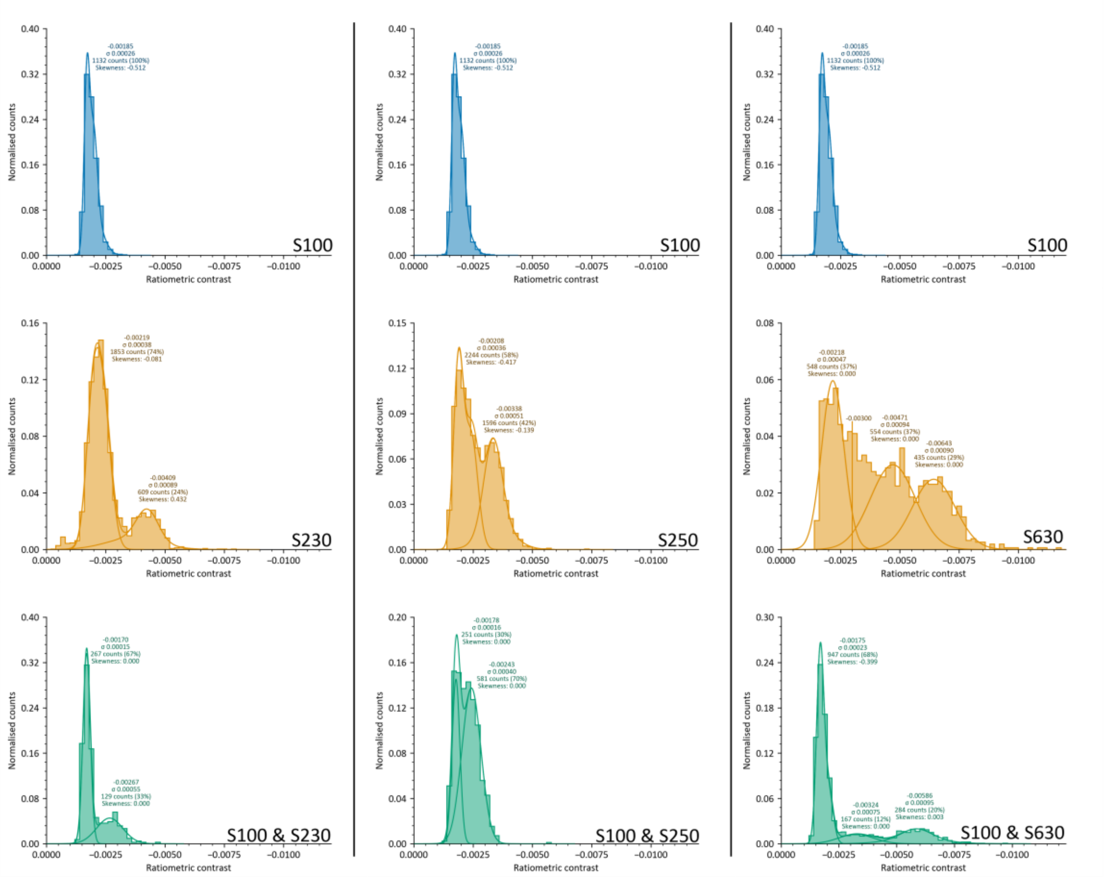

Figure S5. Mixtures of PEO as resolved on MP at 1-7 nM (7.5E-4 mg/mL). The top two rows of each column are mixed to create the bottom row of each column. Large FOV and Buffer Free autofocus were used for MP, 60 seconds of data collected.

Therefore, for successful resolution of mixtures, particularly those with significant differences in mass, equimolar concentrations should be utilized rather than equimass concentrations.

Effects of Increasing Acquisition Time

Lowering the concentrations of analyte used for MP provided better resolution of subpopulations within the MMD of the PEO, but datasets with fewer counts overall result are less statistically robust. Extending the acquisition time of the video is a natural remedy to increase the data set size, as well as to observe more incidences of low-population species. However, the number of detected counts dropped considerably after the first 120 seconds (Figure S6), likely to due irreversible adsorption. This explanation is consistent with the relative dearth of positive contrast events which indicate molecular departure. Thus, the benefits of extending the acquisition time rapidly diminished after the first 60-120 seconds. Additionally, adjusting the acquisition time did not alter the distribution of contrasts measured. As shown in Figure S7, the first 100 events and last 100 events from a 600 second long video taken of S230 are nearly identical, suggesting that there is no timing bias present (i.e. larger polymers tending to land later). Therefore, if the concentration is high enough to observe a sufficient number of events, the acquisition time itself will not affect the distribution of polymers sampled.

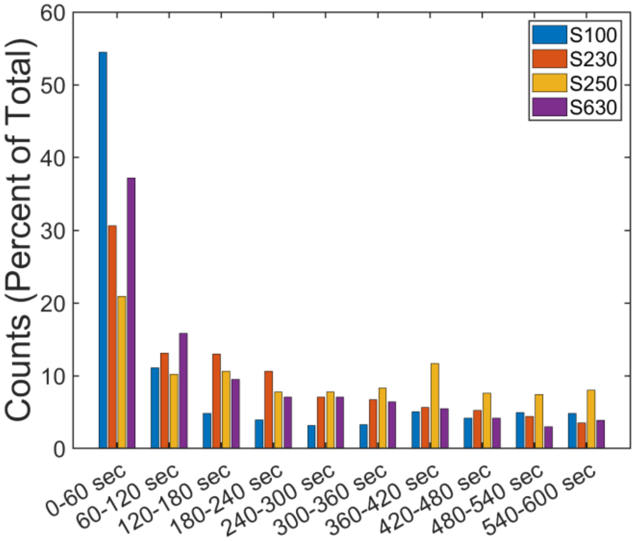

Figure S6. Counts observed over time on mass photometry (LFOV, DDFF)

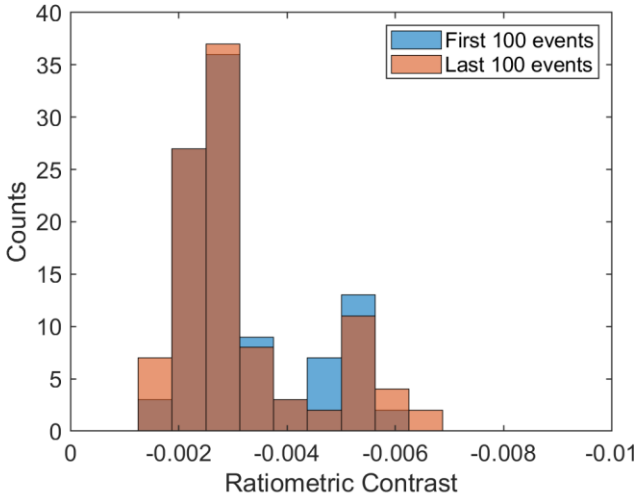

Figure S7. First and last 100 events from a 600 second video taken of S230 (LFOV, DDFF)

Effects of FOV and Autofocus Settings

The Refeyn Two^{MP} has multiple operating parameters. There are two autofocus settings ("Buffer Free Find Focus," BFFF and "Droplet Dilution Find Focus," DDFF). [12] BFFF and DDFF autofocus settings differ in what interface is used to set the focus, either locking focus to a dry coverslip before solution is added (BFFF), or to a coverslip with solvent already in the sample region (DDFF). While both autofocus settings provide accurate measurements of contrast because the focus position can shift slightly between settings, the best practice for an MP experiment is to use the same focus finding method for a mass calibration as the measurements that use that calibration. [12] The instrument also has three field of view settings (FOV; large: 202.4 μm^2, regular: 46.3 μm^2, small: 29.8 μm^2). Unless otherwise stated, the large FOV was used to maximize the number of detected events. At the lowest sample concentrations (~0.25nM), some differences in histogram shape were noticed between DDFF and BFFF modes. These differences may be attributed to issues in achieving well mixed samples since the polymers must be delicately handled to limit scission of the large, fragile chains. Gently but thoroughly mixing the relatively large volume of sample prepared for the BFFF measurement presented a greater practical challenge than mixing the relatively small volume of solution prepared on the slide for the DDFF using a pipette. Additionally, the sample for the BFFF measurement sits in a vial for several minutes at low concentration before measurement while the DDFF sample is measured almost immediately after its preparation. As sample concentration was increased to approximately 1nM and higher, we found that DDFF and BFFF measurements were qualitatively similar.

Discussion of polymer breakdown at the surface in MP

The interaction of the polymer with a glass surface during MP is qualitatively similar to the interaction of a polymer with the stationary phase (often silica) in an SEC experiment. Actually, a key difference is that in SEC, the interaction is occurring under substantially higher pressures and at elevated temperatures as well. Since SEC is routinely used to separate high molecular weight PEO, both analytically and preparatively, it seems unlikely that the surface in MP would be causing substantial chain scission.

Chain scission by itself would not result in an altered MP reading. If a PEO molecule landed on the glass surface and broke into 2 or more components, the mass reading would not change, because those components would all still be present in the same diffraction-limited spot. For a lower mass to be registered, one or more of the components would need then to detach and diffuse into solution (or away on the surface), resulting in a lower mass reading. However, such dynamics are not observed. MP measurements are *differential* measurements: two images are compared, before and immediately after binding. If a fragment were to leave the spot in another frame, this would be observed as a positive contrast peak. Such delayed positive peaks are not observed. In fact, nearly all of the observed signals are negative contrasts, indicative of the macromolecule landing on the surface, not detaching. This suggests that attachment to the surface is largely irreversible. Taken together, we see no evidence for chain scission and substantial evidence that polymers do not return to solution after binding.

Size Exclusion Chromatography

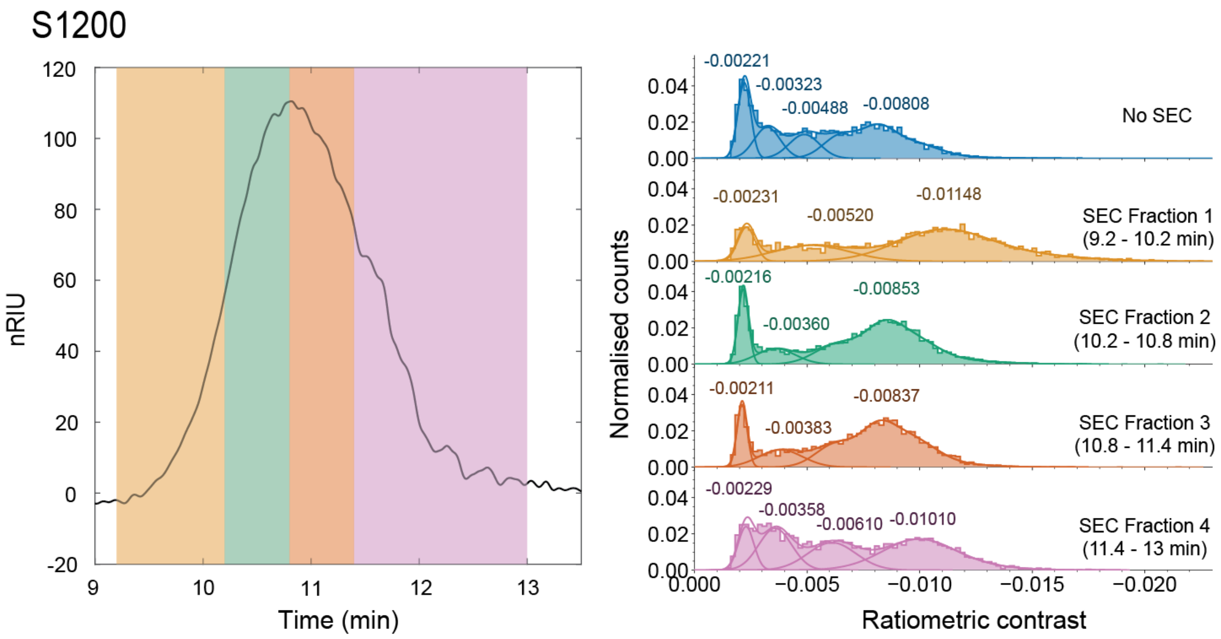

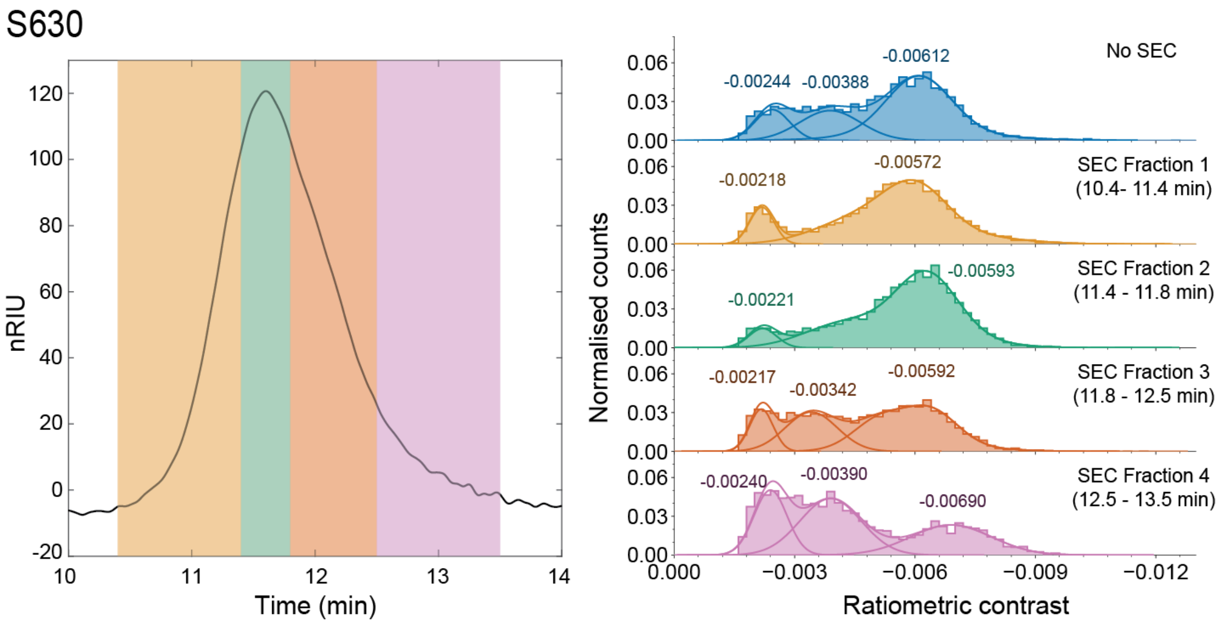

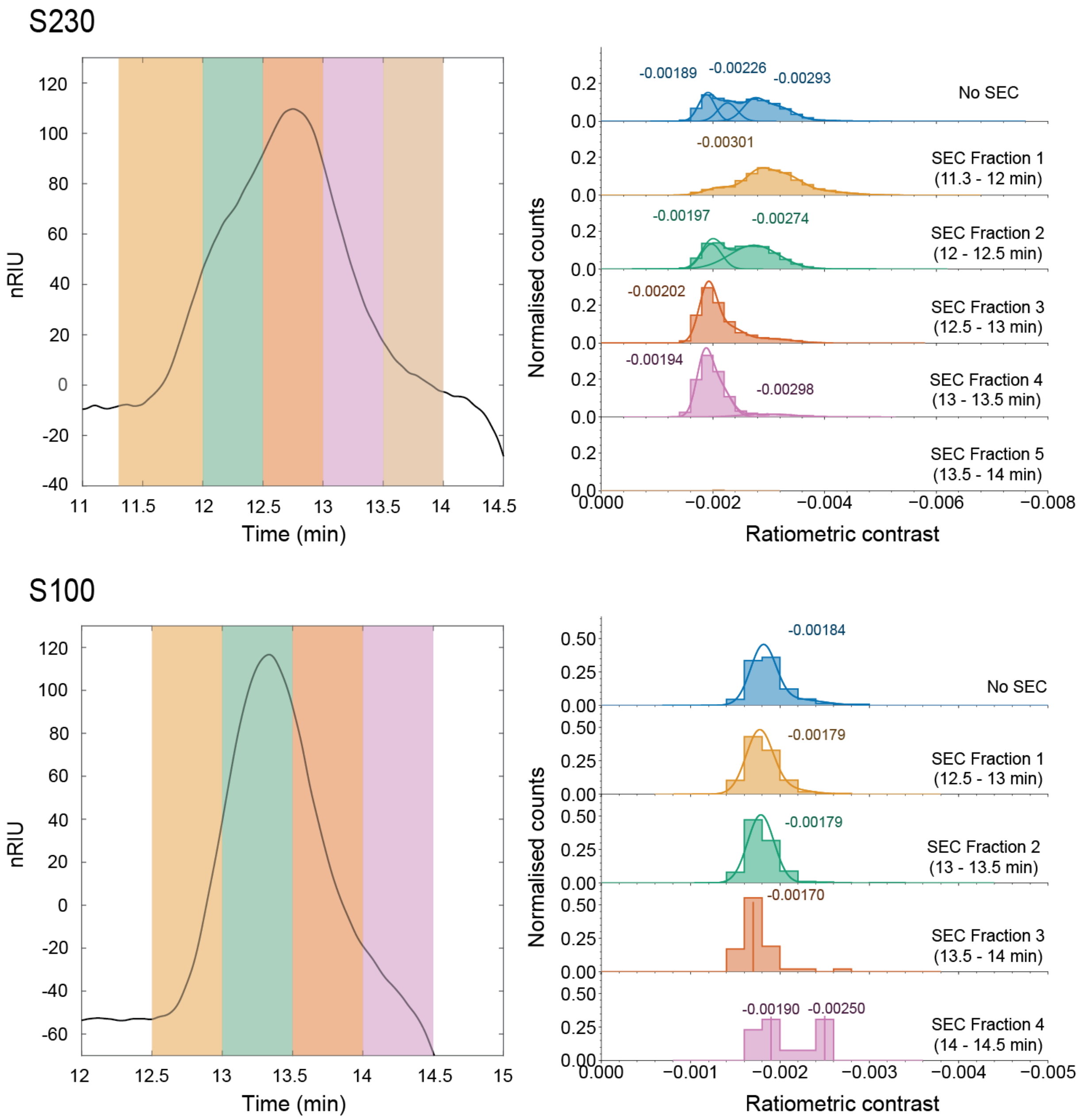

Figure S8. SEC-RID traces of S1200, S630, S230, and S100, with fractions measured on MP after separation.

SEC with RID traces and MP of the collected fractions of each peak are shown in Figure S8 (S1200, S630, S230, and S100) and Figure 5 (S250). As expected, the larger weight samples eluted more quickly and produced broader SEC peaks than the smaller samples. The S250 sample shown in Figure 5 was taken with a slightly shorter length of tubing between the column and RID and therefore has a slightly shorter retention time relative to the samples shown in Figure S8. The MP of all samples except S100 show a

shift in population from larger contrasts toward smaller contrasts as the fraction position moves later in the peak.

The complementary nature of MP and SEC is futher illustrated by the fact that the MP distribution for an unfractionated PEO sample can be reconstructed using a weighted sum of the MP distributions of the individual fractions as shown in Figure S9 for the S250 data presented in Figure 5. The reconstructed (weighted, red) MP probability distribution is created by using the SEC trace, Figure 5 (left), to yield a series of normalized weights, which are then applied to the MP distributions from the individual fractions. This trace is then compared, as a normalized probability distribution, to the "No SEC" trace (blue). The reconstruction faithfully reproduces the multimodal structure, in qualitative agreement with the "No SEC" trace.

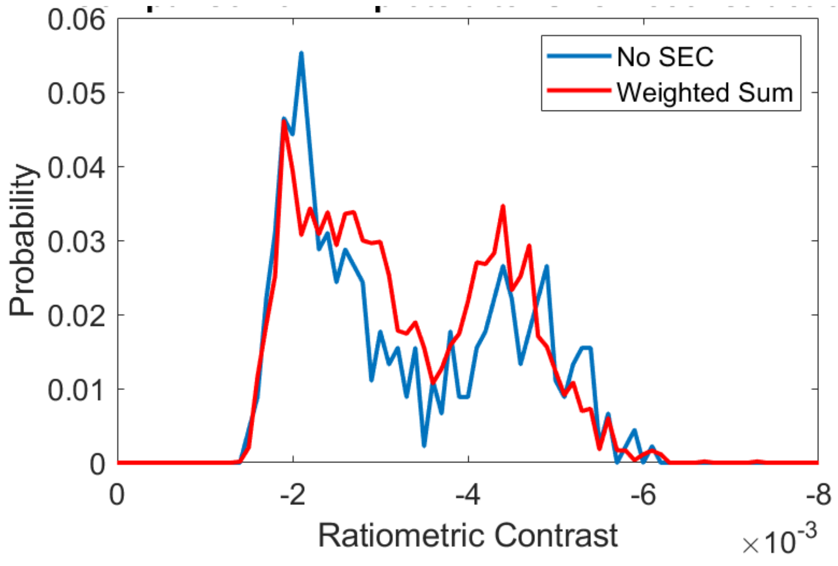

Figure S9. "No SEC" trace from Fig. 5 compared with the reconstructed SEC trace from the fractionated MP histograms

Sonication

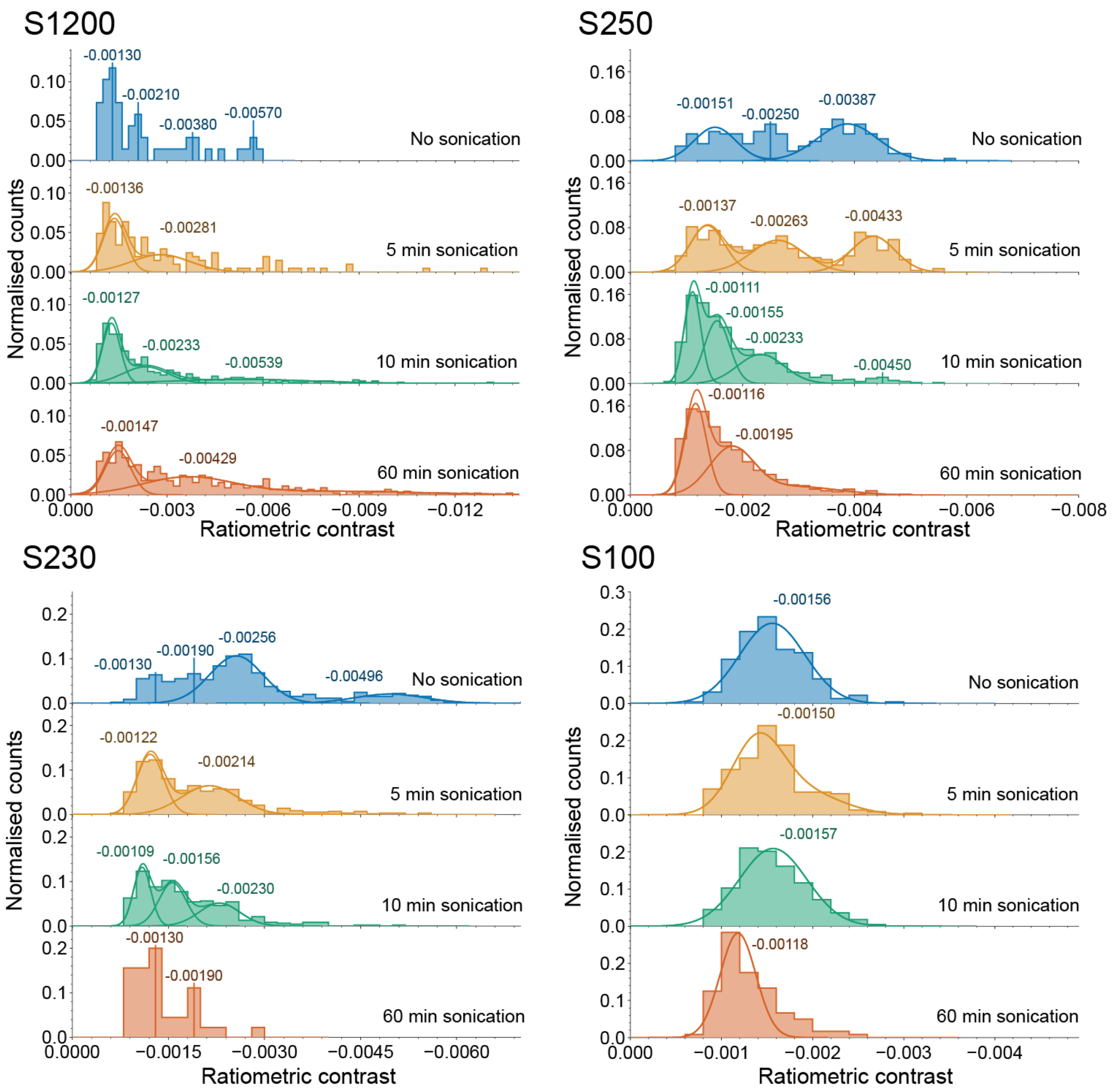

Figure S10. MP histograms of S1200, S250, S230, and S100 sonicated for 0, 5, 10, and 60 minutes.

The effects of sonication on the different PEO standards varied with the polymers' size. The larger the polymer chain, the faster the disappearance of its highest-mass populations. Figure S10 is a supplement to Figure 6 in the main text, showing the sonicated behavior of S1200, S250, S230, and S100 after 0, 5, 10, and 60 minutes of sonication. Unlike the data in Figure 6, the data in Figure S10 is taken with the small FOV,

which, while better for resolving low mass objects can struggle to resolve higher mass species (unsonicated S1200).

Unlike the higher mass standards, S100 was minimally affected by sonication. Although the peak ratiometric contrast did shift to a slightly lower value over the hour of sonication, the data was still well-fit by a single Gaussian, suggesting that the smaller chains were less susceptible to sonication-induced chain scission. However, the number of counts observed between 10 and 60 minutes of sonication did drop by around 50% which is potentially indicative of some breakdown to pieces smaller than the MP can reliably measure, as S100 is close to the detection threshold for PEO.

Sonication and Size Exclusion Chromatography

In an attempt to further investigate sonication induced chain scission, SEC-RID followed by fractionation and MP was performed on samples of S230 sonicated for 0, 10 and 60 minutes (Figure S11). As sonication time increases, the SEC-RID peak noticibly broadens and shifts toward longer elution times, suggesting significant breakdown of S230 chains during sonication. As discussed above with Figure S10 and in main text Figure 5, MP of earlier timepoint fractions has larger proportions of large contrast events while later fractions have mainly smaller contrast events. Notably, late fractions in the sample sonicated for 60 minutes are far more populated than the late fractions for the non-sonicated sample.

An interesting phenomenon most apparent in the data from 60 minutes of sonication is that the MP contrast ratios of the fractions collected from the size exclusion column do not sum up to the MP contrast ratios of the non-fractionated sample. The non-fractionated sample was prepared by making a 2 mg/mL solution of S230 and sonicating it for an hour, then diluting this solution to a concentration of 0.5 nM for MP based on the assumed starting concentration of 2 mg/mL. This polymer solution can be considered as a mixture of species, and while the mass per volume concentration was consistent over the course of the sonication, the molarity of larger species decreased over the course of the sonication, while the molarity of smaller species increased as polymer chains broke. Since each SEC fraction is diluted to an approximate concentration of 0.5 nM for MP, the concentration of a particular sized species in each fraction may be much higher relative to the concentration of the same species in the unfractionated sample. The lack of events observed in MP for the non-fractionated S630 after 60-minute sonication may be due to the breakdown of the majority of chains with molecular weights high enough to be detectable by MP. The relatively small number of remaining larger species are more easily measured by MP in the fractionated samples because they have a sufficiently high concentration for MP detection.

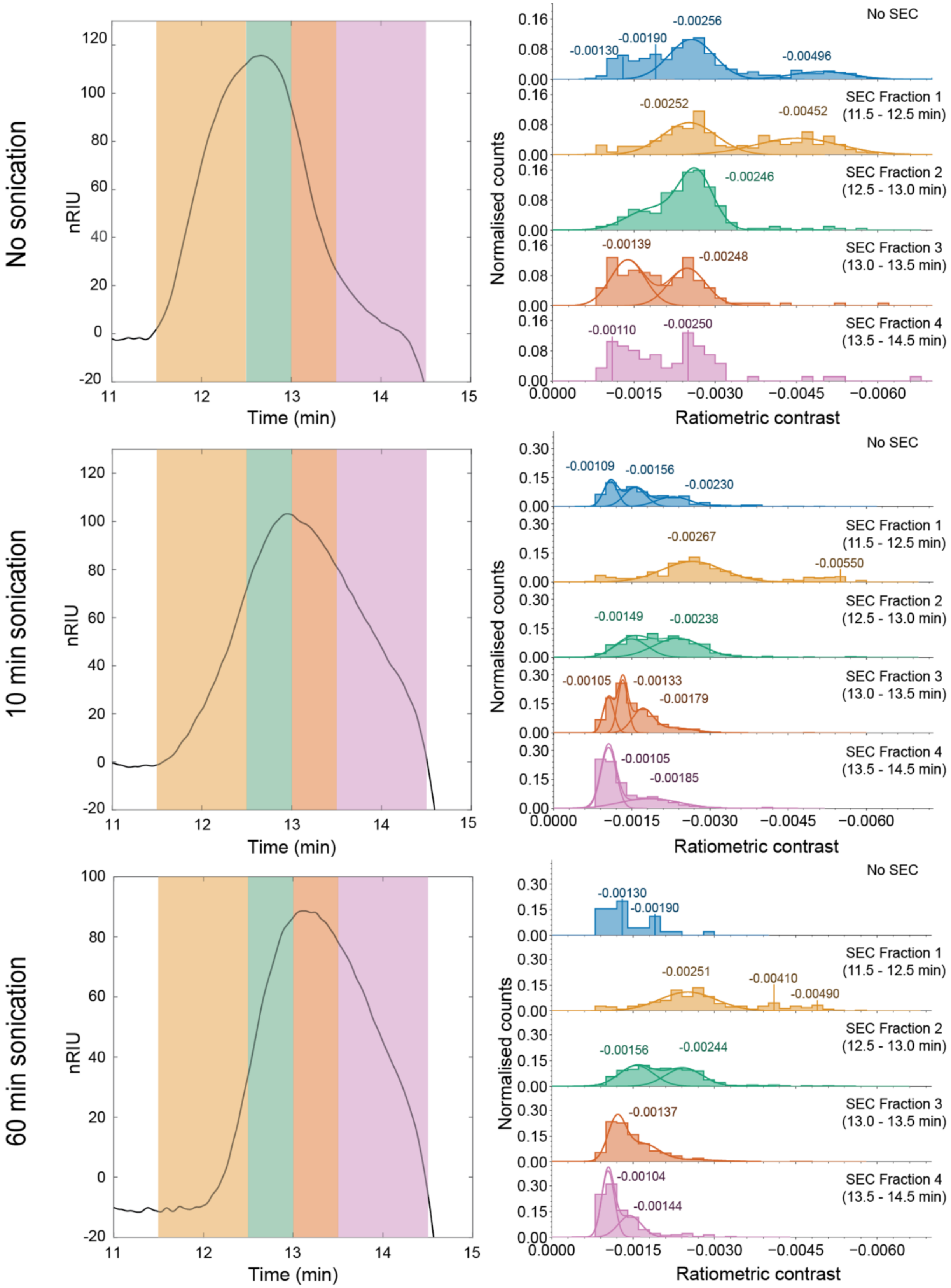

Figure S11. SEC (left) and MP of SEC fractions (right) of S230 sonicated for 0 (top), 10 (middle) and 60 minutes (bottom).

Stability of Standards

As described in the Experimental section, PEO was allowed to dissolve overnight undisturbed at room temperature and samples were mixed via gentle swirling of the vial to minimize chain scission. However, it is possible that some sample degradation occurred durng storage of the sample, which was maintained at room temperature. To test this hypothesis, a second sample of S250 was obtained from the manufacturer with the same lot number and immediately analyzed. The histograms of contrast values for the two samples (Figure S12) are nearly identical, indicating that substantial sample degradation was not experienced during room temperature storage.

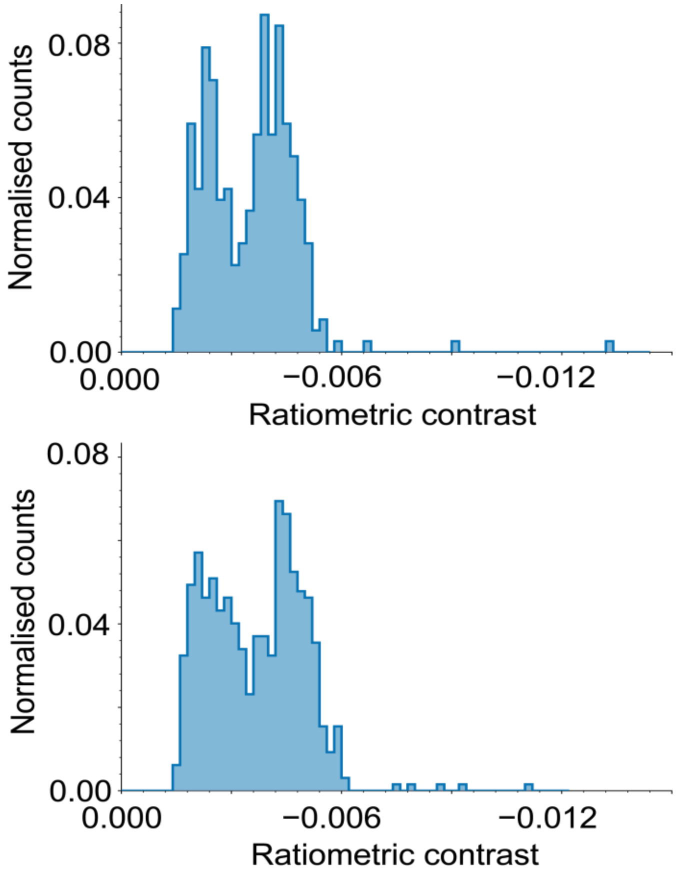

Figure S12. MP data showing histograms of contrast values for two samples of S250 from the same manufacturer lot. The upper histogram was from a sample stored at room temperature for several months with data acquired from aliquots throughout this window. The lower histogram was analyzed right after it was received from the manufacturer.

Measurement Reproducibility

Figure S13 shown below contains 3 individual histograms each for S250, S230, and S100. All data were collected on the same day, and each movie was taken of an aliquot from the same stock of each PEO sample. The bottom row is data shown in Figure 2 reproduced here for ease of comparison. The shape of all three histograms for each of the samples is nearly identical. As this data was all taken within a 2 hour period, the contrast values are also remarkably consistent from run to run of the same sample. If instead these data had been collected over a longer period or several days, we would not expect the contrast values to be identical due to instrumental drift and differences in background intensity. However, even as contrast values shift, the shape of the histograms

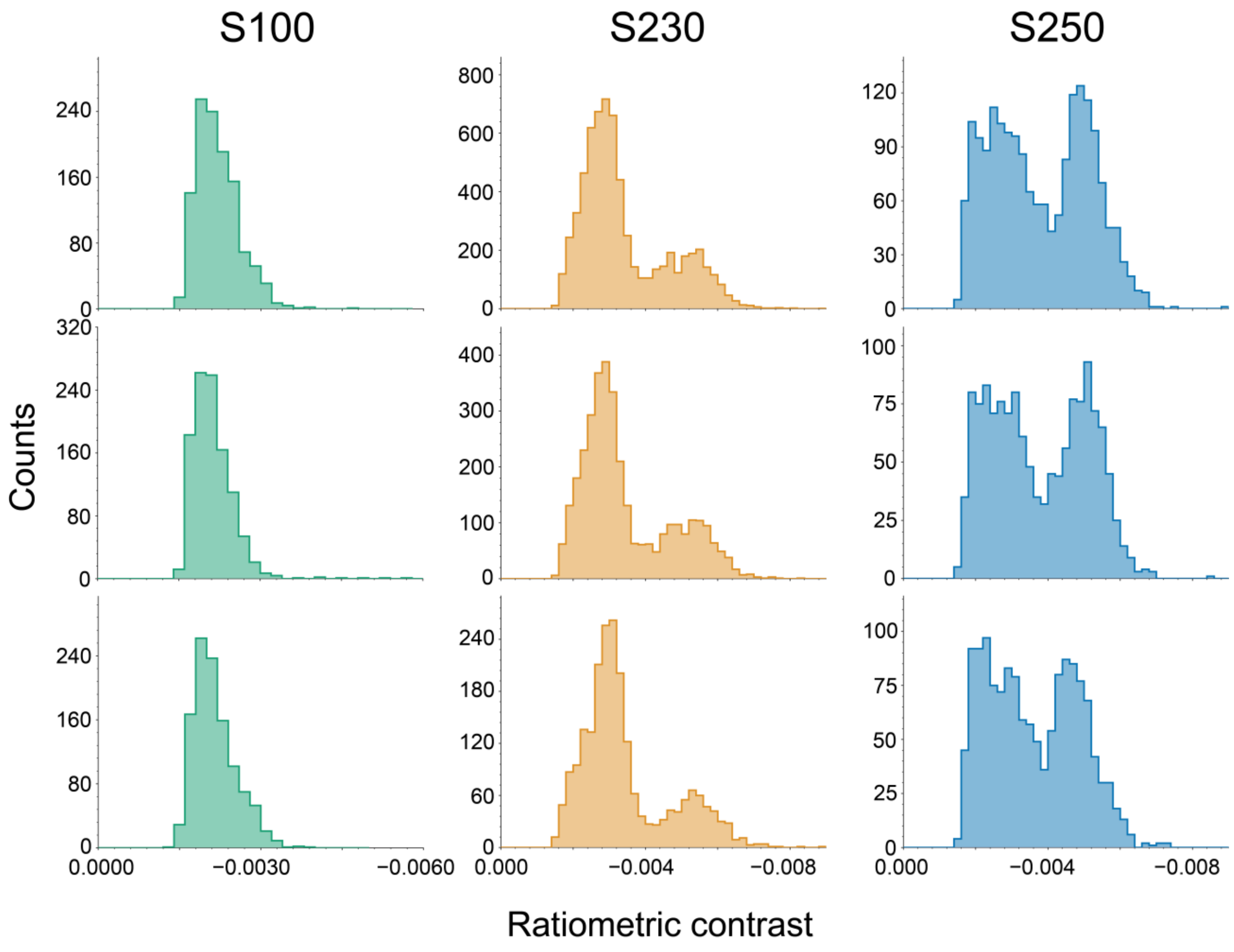

Figure S13. MP data showing histograms of contrast values for S100 (green), S230 (yellow), and S250 (blue). Each histogram is constructed from a single 120s MP movie and all data were taken at 0.7 nM with DDFF and the large FOV.

remains consistent for each sample as long as measurement settings and approximate concentration are the same. Comparison of the histograms shown in Figure S13 to those shown in main text Figure 4 illustrates the reproducibility of the histogram shape across different days. Minor differences in histogram shape between Figure S13 and Figure 4 can be attributed to the macroscopic heterogeneity existing across different sample granules which results in the slight differences in MMD observed.

Sensitivity in Non-polar Solvents

The excess polarizability, α, is a function[7] of the refractive indices of a scatterer, n_p and a medium, n_m:

$$\alpha \propto \frac{n_p^2 - n_m^2}{n_p^2 + 2n_m^2}$$

Given some refractive indices of known materials (n_{PEO}=1.45, $n_{Protein}$=1.48, n_{PS}=1.59, n_{PP}=1.47) and solvents (n_{water}=1.45, $n_{methylethylketone}$=1.37, $n_{toluene}$=1.50), one can calculate factors for different analyte/solvent combinations:

Protein in water	0.074
PEO in water	0.059
Polystyrene (PS) in methy ethyl ketone (MEK)	0.104
PS in toluene	0.040
Polypropylene (PP) in toluene	-0.013

The limit of detection for MP for proteins in water is around 30kD,[3] though down to 10kD is achievable with further analysis via machine learning.[13] The limit of detection should scale roughly inversely with the excess polarizability. A back of the envelope calculation then suggests that the limit of detection for PEO in water should be around 40kD, similar to what is observed. Further analysis suggests that the limit of detection for PS in MEK (or acetone or ethyl acetate, which have similar refractive indices) should be improved, since the refractive index contrast is large, around 20kD. But, PS in toluene, where there will be a lower refractive index contrast, should exhibit a worse limit of detection, around 55kD. PP in toluene is nearly a worst case scenario, where the polymer and solvent are nearly index matched, with predicted limit of detection of 170kD. Here, use of a highly chlorinated solvent, like trichlorobenzene, with a refractive index of 1.57, where the medium actually has a higher index than the molecule, would likely result in improved contrast. In general, the largest index contrast between polymer and medium is expected to yield the lowest limit of detection. Higher refractive index solvents may also reduce speckling in the image, and so may do better than this back of the envelope calculation.

Increased contrast can also potentially be obtained through use of a physical substrate (cover slip) with higher refractive index.[7]

Histogram Counts and Fitting Quality

Figure	Counts	Figure	Counts
2a	1731	4 (S100 & S250)	645
2b	1920	4 (S100 & S630)	395
2c	1437	5 (No SEC)	452
2d	2185	5 (Fraction 1)	1302
2e	1109	5 (Fraction 2)	419
2f	362	5 (Fraction 3)	1608
4 (S100)	177	5 (Fraction 4)	206
4 (S230)	300	6 (No sonication)	1706
4 (S250)	335	6 (5 minutes)	3008
4 (S630)	604	6 (10 minutes)	4653
4 (S100 & S230)	445	6 (60 minutes)	2952

Table S1. Table displaying total number of counts in each of the main text MP histograms.

Sample	Gaussian Peak Contrast	Fit Error (x 10^{-5})
S100	-0.00204	2.41
S230	-0.00258	3.17
	-0.00495	6.01
S250	-0.00230	3.39
	-0.00418	4.03
S630	-0.00221	3.82
	-0.00445	9.21
	-0.00761	4.82
S100 & S230	-0.00248	2.12
	-0.00480	9.08
S100 & S250	-0.00210	1.70
	-0.00433	3.98
S100 & S630	-0.00203	2.44
	-0.00798	7.12

Table S2. Table displaying the fit error for the Gaussian fits shown in Figure 4. Fit error is calculated by dividing the full width at half maximum of each Gaussian by the square root of the number of counts associated with that Gaussian fit.

[1] R. W. Taylor, V. Sandoghdar, *Nano Letters* **2019**, *19*, 4827-4835.
[2] G. Young, P. Kukura, *Annual Review of Physical Chemistry* **2019**, *70*, 301-322.
[3] G. Young, N. Hundt, D. Cole, A. Fineberg, J. Andrecka, A. Tyler, A. Olerinyova, A. Ansari, E. G. Marklund, M. P. Collier, S. A. Chandler, O. Tkachenko, J. Allen, M. Crispin, N. Billington, Y. Takagi, J. R. Sellers, C. Eichmann, P. Selenko, L. Frey, R. Riek, M. R. Galpin, W. B. Struwe, J. L. P. Benesch, P. Kukura, *Science* **2018**, *360*, 423-427.
[4] D. Cole, G. Young, A. Weigel, A. Sebesta, P. Kukura, *ACS photonics* **2017**, *4*, 211-216.
[5] N. S. Ginsberg, C.-L. Hsieh, P. Kukura, M. Piliarik, V. Sandoghdar, *Nature Reviews Methods Primers* **2025**, *5*, 23.
[6] R. Asor, D. Loewenthal, R. van Wee, J. L. Benesch, P. Kukura, *Ann Rev Biophys* **2025**, *54*, 379-399.
[7] J. Becker, J. S. Peters, I. Crooks, S. Helmi, M. Synakewicz, B. Schuler, P. Kukura, *ACS photonics* **2023**, *10*, 2699-2710.
[8] F. Vollmer, D. Braun, A. Libchaber, M. Khoshsima, I. Teraoka, S. Arnold, *Appl Phys Lett* **2002**, *80*, 4057-4059.
[9] F. Bailey Jr, R. Callard, *Journal of applied polymer science* **1959**, *1*, 56-62.
[10] R. Barer, S. Joseph, *Journal of Cell Science* **1954**, *s3-95*, 399-423.
[11] P. Kratochvil, D. Strakova, J. Stejskal, Z. Tuzar, *Macromolecules* **1983**, *16*, 1136-1143.
[12] RefeynLtd, *Refeyne TwoMP Mass Photometer User Manual Version 1.0*, **2024**.
[13] M. Dahmardeh, H. Mirzaalian Dastjerdi, H. Mazal, H. Köstler, V. Sandoghdar, *Nat Methods* **2023**, *20*, 442-447.
